# Study on potential markers for diagnosis of renal cell carcinoma by serum untargeted metabolomics based on UPLC-MS/MS

**DOI:** 10.3389/fphys.2022.996248

**Published:** 2022-11-29

**Authors:** Jun Wang, Wen-Yu Yang, Xiao-Han Li, Bei Xu, Yu-Wei Yang, Bin Zhang, Chun-Mei Dai, Jia-Fu Feng

**Affiliations:** ^1^ College of Medical Technology, Chengdu University of Traditional Chinese Medicine, Chengdu, China; ^2^ Department of Medical Laboratory, Affiliated Hospital of Southwest Medical University, Luzhou, China; ^3^ Department of Clinical Laboratory, Mianyang Central Hospital, School of Medicine, University of Electronic Science and Technology of China, Mianyang, China

**Keywords:** renal cell carcinoma, benign kidney tumor, untargeted metabolomics, lipid metabolism, amino acid metabolism, potential biomarkers

## Abstract

**Objective:** Renal cell carcinoma (RCC) is the most common malignancy of the kidney. However, there is no reliable biomarker with high sensitivity and specificity for diagnosis and differential diagnosis. This study aims to analyze serum metabolite profile of patients with RCC and screen for potential diagnostic biomarkers.

**Methods:** Forty-five healthy controls (HC), 40 patients with benign kidney tumor (BKT) and 46 patients with RCC were enrolled in this study. Serum metabolites were detected by ultra-high performance liquid chromatography-tandem mass spectrometry (UPLC-MS/MS), and then subjected to multivariate statistical analysis, metabolic pathway analysis and diagnostic performance evaluation.

**Results:** The changes of glycerophospholipid metabolism, phosphatidylinositol signaling system, glycerolipid metabolism, d-glutamine and d-glutamate metabolism, galactose metabolism, and folate biosynthesis were observed in RCC group. Two hundred and forty differential metabolites were screened between RCC and HC groups, and 64 differential metabolites were screened between RCC and BKT groups. Among them, 4 differential metabolites, including 3-β-D-Galactosyl-sn-glycerol, 7,8-Dihydroneopterin, lysophosphatidylcholine (LPC) 19:2, and γ-Aminobutyryl-lysine (an amino acid metabolite), were of high clinical value not only in the diagnosis of RCC (RCC group vs. HC group; AUC = 0.990, 0.916, 0.909, and 0.962; Sensitivity = 97.73%, 97.73%, 93.18%, and 86.36%; Specificity = 100.00%, 73.33%, 80.00%, and 95.56%), but also in the differential diagnosis of benign and malignant kidney tumors (RCC group vs. BKT group; AUC = 0.989, 0.941, 0.845 and 0.981; Sensitivity = 93.33%, 93.33%, 77.27% and 93.33%; Specificity = 100.00%, 84.21%, 78.38% and 92.11%).

**Conclusion:** The occurrence of RCC may involve changes in multiple metabolic pathways. The 3-β-D-Galactosyl-sn-glycerol, 7,8-Dihydroneopterin, LPC 19:2 and γ-Aminobutyryl-lysine may be potential biomarkers for the diagnosis or differential diagnosis of RCC.

## 1 Introduction

Kidney carcinoma is one of the most common malignancies of the urinary system, and renal cell carcinoma (RCC) accounted for more than 85% of kidney carcinoma (Nabi et al., 2018). According to the GLOBOCAN 2020 reports, there are more than 430,000 new cases of RCC and nearly 180,000 new deaths annually worldwide (Sung et al., 2021). In the early stages of RCC, patients are usually asymptomatic, but RCC patients’ conditions usually progress to the intermediate or advanced stage, when they present with the typical classic clinical triad of gross hematuria, flank pain, and palpable abdominal mass (Gray and Harris, 2019). Although modern imaging techniques are widely available to detect most kidney masses, 20%–30% of patients with RCC have metastases at diagnosis (Petejova and Martinek, 2016). Moreover, most patients with RCC are almost always detected by brightness-mode ultrasound, computed tomography, or magnetic resonance imaging when screening for other diseases not related to the kidney (Capitanio et al., 2019). Therefore, early diagnosis of RCC is essential to improve treatment outcomes and reduce mortality in patients with RCC.

The commonly used kidney function indicators in clinic, such as urea, creatinine (Cr), uric acid (UA), cystatin C (CysC), complement C1q (C1q), neutrophil gelatinase-associated lipocalin (NGAL), and estimated glomerular filtration rate (eGFR), can evaluate the kidney function of patients with RCC, but cannot indicate kidney tumors. To date, few reliable tumor-specific markers are available for clinical use in kidney carcinoma. Although kidney biopsy is the "gold standard” for the diagnosis and identification of kidney tumors, its clinical application is often limited because of its invasive nature and the possible risk of needle tract implantation and metastasis.

In recent years, metabolomics technology has been widely used to screen for potential biomarkers of diseases, especially tumors, and to explore the occurrence and development of diseases through the metabolic pathways of substances *in vivo* (Wang et al., 2021a). Some early studies investigated the urinary metabolomics of patients with RCC using ultra-high performance liquid chromatography-tandem mass spectrometry (UPLC-MS/MS) and found a metabolite panel consisting of cortolone, testosterone and L-2-aminoadipate adenylate, and that combined detection of L-3-hydroxykynurenine, 1,7-dimethylguanosine and Tetrahydroaldosterone-3-glucuronide may be used for distinguishing RCC from benign kidney tumor (BKT) (Liu et al., 2019a; Zhang et al., 2020a). Another study confirmed that analysis using blood samples could more accurately reflect the metabolic changes in tumor tissues than using urine samples (Ganti et al., 2012). Additionally, some scholars found by plasma metabolomics (Liu et al., 2020) that combined detection of diaminopimelic acid, 12,13-DHOME, 5-L-glutamyl-L-alanine, PC (38:4), 4,8- dimethylnonanoyl carnitine and cholesteryl 11-hydroperoxy-eicosatetraenoate could be used for the differential diagnosis of benign and malignant kidney tumors. However, these screened potential biomarkers had low diagnostic performance, sensitivity and specificity for identifying RCC. Furthermore, it has been shown that the metabolomic analysis of serum samples is more reliable than plasma samples (Yu et al., 2011; Sotelo-Orozco et al., 2021).

Lipids are important components in serum, and a study found that disturbed lipid metabolism was strongly associated with disease severity of RCC and may be a risk factor for RCC development (Li et al., 2020). Based on the above theories and research findings, we propose a hypothesis: compared with healthy subjects or BKT, RCC patients have abnormal metabolic pathways of some substances. In this study, we analyzed the serum metabolomic profile of patients with RCC and BKT by untargeted metabolomics based on UPLC-MS/MS. We also investigated the relationship of metabolites with common indicators for kidney function and lipid indicators. The results of this study may provide new insights into the pathogenesis of RCC and provide evidence for screening new potential biomarkers for its diagnosis and differential diagnosis.

## 2 Materials and methods

### 2.1 Subjects

Between March 2021 and March 2022, 86 patients with kidney tumors, including 46 cases with RCC and 40 cases with BKT, who were admitted to the Department of Urology, Mianyang Central Hospital were enrolled in the study.

Inclusion criteria: 1) age ≥18 years; 2) All patients were diagnosed as RCC or BKT by pathological biopsy after finding abnormalities in computed tomography (CT) or magnetic resonance imaging (MRI) examination. Exclusion criteria: 1) patients with failure of blood sample collection; 2) women in pregnancy or lactation; 3) patients complicated with other metabolic diseases and tumors; 4) patients with RCC who underwent treatment with radiotherapy or immunotherapy before enrollment; 5) patients who had undergone nephrectomy. In addition, all diagnoses were confirmed by senior clinicians according to clinical diagnostic criteria.

Forty-five healthy subjects who underwent physical examination during the same period were enrolled as healthy controls (HC). Inclusion criteria: 1) age≥18 years; 2) healthy volunteers with normal examination indexes during the same period; 3) subjects who did not take drugs that may affect kidney function within the last month. Exclusion criteria: 1) patients with failure of blood sample collection; 2) women in pregnancy or lactation.

Detailed clinical information of the subjects is shown in Supplementary Table S1. This study was approved by the Medical Ethics Committee of Mianyang Central Hospital (P2020030), and all patients signed the informed consent.

### 2.2 Sample collection

After fasting overnight, all study subjects were subjected to blood collection (approximately 5.0 ml of venous blood each) from 8:00–10:00 a.m. The SSTTM II Advance vacuum blood collection tube (BD Vacutainer®, United States) containing separation gel and coagulant was used. After centrifugation at 3,000 *g* for 10 min, the serum was collected and equally divided into two parts. One part was tested for kidney function and lipid indicators within 2 h. The other part was stored at −80°C for metabolomics analysis.

### 2.3 Detection of common kidney function and lipid indicators

The indicators for kidney function and lipid indicators were measured on LST008 automatic biochemical analyzer (Hitachi, Japan). The C1q kit was provided by Shanghai Beika Biochemical Reagent Co., Ltd. (Shanghai, China). The kits for other indicators were provided by Sichuan Maker Biotechnology Co., Ltd. (Sichuan, China).

### 2.4 Ultra-high performance liquid chromatography-tandem mass spectrometry analysis

#### 2.4.1 Sample preparation and analysis

A mixture of 190 µl of serum sample, 10 µl of internal standard (10 μg/ml, clenbuterol and chloramphenicol mixture) and 800 µl of methanol-acetonitrile (v/v = 1:1) solution was sonicated at 4°C for 10 min, then the mixture was incubated at –20°C for 1 h, followed by centrifugation at 13,000 *g* for 15 min at 4°C to obtain the supernatant. The supernatant was filtered by 0.22 µm microporous membrane. Finally, 3 µl of the filtrate-solution was transferred by an autosampler and injected into the UPLC-MS/MS system for metabolomic analysis. In addition, 10 µl of serum from each sample was mixed as a quality control (QC) sample. QC samples were processed in the same way as the study samples. Serum metabolomics analysis was performed with Agilent^®^ 1290 Infinity II UPLC system (Agilent Technologies Inc., United States) and AB Sciex^®^ Triple TOF 5600^+^ mass spectrometer system (AB Sciex, United States). UPLC-MS/MS analytical conditions used previous methods of our lab (Xu et al., 2021). In addition, QC samples were tested after every 10 samples in the analysis sequence to evaluate the reliability of the large-scale metabolomics analysis and the stability of the instrument (Dudka et al., 2020; Zhu et al., 2021).

#### 2.4.2 Metabolite identification and analysis

Raw data were collected using Analyst TF 1.7 software (AB Sciex, United States of America). The metabolomics data were subjected to a series of processing workflow, including peak picking, quality assessment, missing value imputation, normalization, transformation and scaling. The details were as follows: 1) the XCMS algorithm was applied to peak extraction based on the One-MAP platform (http://www.5omics.com) provided by Dalian Dashuo Information Technology Co. (Dalian, China). 2) quality analysis of the data based on the stability of the QC samples. The percentage of relative standard deviation (RSD) of metabolic mass spectrometry features in QC samples that were less than 50% should exceed 80%. Calibration of QC was performed using the statTarget analysis (Luan et al., 2018). 3) the 80% rule was used to exclude metabolic features with more than 20% of non-zero values in any category of samples in the metabolic features. Missing values were filled with the smallest value in the variable. 4) normalization of data was performed using the MetNormalizer method of QC-based support vector regression analysis and the internal standards was used for checking the stability of the instrument performance. 5) the feature variables were processed by auto scaling in principal component analysis (PCA) and partial least squares analysis (PLS-DA). This was to eliminate differences in the order of magnitude of the concentration of different metabolites. The characteristic peaks of metabolites were carried out by molecular weight error (<20 ppm), signal-to-noise ratio and summation ions to predict their molecular formulae. Metabolite identification was annotated by scoring each peak based on matches to accurate masses, retention times, and MS/MS fragmentation to the standard compounds databases (containing information of 1,550 metabolic standards), and custom databases including METLIN (http://metlin.scripps.edu/), Kyoto Encyclopedia of Genes and Genomes (KEGG) (http://www.kegg.jp/kegg/pathway.html), LipidMaps (https://www.lipidmaps.org/), Human Metabolome Database (HMDB) (https://hmdb.ca/), MassBank (https://massbank.eu/), and PubChem Database (https://pubchem.ncbi.nlm.nih.gov/), parameters setting: Δm/z (MS1)≤0.01000Da; Δm/z (MS2)≤0.05000Da; MS2 Score Method = Forward; Reference Noise of Unknown MS2 to remove = 1.000000; Reference Noie of Standard MS2 to remove = 200.000000; the number of near fragments at least to merge peaks cluster = 2; investigate the maximum number of fragments = 2. According to the formal definition of metabolite annotation and identification specified by the Chemical Analysis Working Group of the Metabolomics Standards Initiative (MSI), the metabolites determined in this study would be considered as putative identification (levels 2) (Viant et al., 2017). Untargeted metabolomics does not need a reference standard, but structural information was obtained using MS/MS data and combined with mass-to-charge ratio (m/z) and retention time. Metabolic pathways of the differential metabolites were analyzed by the Kyoto Encyclopedia of Genes and Genomes (http://www.genome.jp/kegg) and MetaboAnalyst database exploration (http://www.metaboanalyst.ca/).

### 2.5 Statistical analysis

Statistical analysis was performed using SPSS 26.0 software (International Business Machines Corp., United States). Normally distributed data were expressed as mean ± standard deviation, and compared with one-way ANOVA followed by LSD test. Non-normally distributed data were expressed as median (interquartile range), and analyzed with Kruskal–Wallis H test. Count data were compared using chi-square test. Spearman correlation was used for correlation analysis. PCA and PLS-DA were performed using SIMCA 14.1 software (Umetrics AB, Umea, Sweden). The validity of the PLS-DA model was examined using a random permutation test (100 times). Receiver operating characteristic curve (ROC) analysis was used to evaluate the diagnostic performance of the differential metabolites. When AUC = 0.50–0.59, 0.60–0.69, 0.70–0.79, 0.80–0.89, or ≥0.90, it means that the diagnostic performance is fail, poor, fair, good, or excellent, respectively (Nahm, 2022). *p* < 0.05 indicated that the difference was statistically significant.

## 3 Results

### 3.1 Kidney function and lipid indicators

As shown in Table 1, the kidney function and lipid indicators of NGAL, Cr, CysC, eGFR, triglyceride (TG), low density lipoprotein cholesterol (LDL-C) and apolipoprotein B (Apo-B) were statistically significant among the HC, RCC and BKT groups (all *p* < 0.05). Pairwise comparison analysis showed that compared with the HC group, NGAL (z = 3.542, *p* < 0.001), Cr (z = 3.352, *p* = 0.001), CysC (z = 3.511, *p* < 0.001), TG (z = 3.521, *p* < 0.001), LDL-C (z = 2.650, *p* = 0.008) and Apo-B (t = 0.368, *p* < 0.001) were significantly increased in the RCC group, whereas eGFR (t = 3.443, *p* = 0.001) was significantly decreased in the RCC group. NGAL (z = 2.705, *p* = 0.007), TG (z = 2.420, *p* = 0.016), LDL-C (z = 2.650, *p* = 0.008) and Apo-B (t = 0.240, *p* = 0.018) were significantly increased in the BKT group than in the HC group. Compared with the BKT group, Cr (z = 2.250, *p* = 0.024) and CysC (z = 2.561, *p* = 0.010) were significantly increased in the RCC group, while eGFR (t = 2.631, *p* = 0.010) was significantly decreased in the RCC group. These results showed that kidney function and lipid indicators were abnormal in patients with RCC to different degrees.

**TABLE 1:** Common renal function and lipid indicators of the study subjects.

Group	HC (*n* = 45)	BKT (*n* = 40)	RCC (*n* = 46)	χ2/F, *P*
UA (μmol/L)	332.0 (269.3, 371.7)	282.4 (229.0, 350.8)	333.3 (236.4, 417.1)	4.354^a^, 0.113
C1q (mg/L)	207.0 (175.5, 235.5)	193.5 (168.4, 229.0)	191.5 (166.6, 221.8)	1.366^a^, 0.505
NGAL (μg/L)	95.0 (78.5, 113.0)	124.0 (85.3, 180.0) *	130.1 (93.0, 164.8) *	13.799^a^, 0.001
Urea (mmol/L)	5.10 (4.13, 5.64)	5.37 (4.07, 6.60)	5.43 (4.46, 6.59)	4.215^a^, 0.122
Cr (μmol/L)	66.0 (53.4, 78.0)	65.6 (54.2, 87.6)	80.1 (61.7, 105.7) *	11.775^a^, 0.003
CysC (mg/L)	0.91 (0.79, 1.00)	0.92 (0.76, 1.18)	1.07 (0.90, 1.26) *	13.391^a^, 0.001
eGFR (ml/min/1.73m^2^)	88.59 ± 12.29	85.75 ± 24.14	73.09 ± 20.85 *	8.057^b^, 0.001
TC (mmol/L)	4.58 ± 0.59	4.89 ± 1.11	5.17 ± 1.37 *	3.297^b^, 0.040
TG (mmol/L)	0.98 (0.87, 1.19)	1.29 (0.99, 2.37) *	1.39 (0.98, 1.97) *	13.261^a^, 0.001
HDL-C (mmol/L)	1.38 (1.14, 1.55)	1.31 (1.17, 1.64)	1.22 (1.05, 1.45)	2.717^a^, 0.257
LDL-C (mmol/L)	2.60 (2.27, 2.96)	3.13 (2.26, 3.56) *	2.99 (2.32, 4.31) *	9.355^a^, 0.009
Apo-A1 (g/L)	1.55 ± 0.21	1.53 ± 0.31	1.48 ± 0.27	0.834^b^, 0.437
Apo-B (g/L)	0.81 ± 0.14	0.93 ± 0.24*	1.00 ± 0.30*	7.263^b^, 0.001

Note: n, case; a is the χ2 value; b is the value of F; HC, healthy control; BKT, benign kidney tumor; RCC, renal cell carcinoma; Cr, creatinine; UA, uric acid; CysC, cystatin C; C1q, complement C1q; NGAL, neutrophil gelatinase-associated lipocalin; eGFR, estimated glomerular filtration rate; TC, total cholesterol; TG, triglyceride; HDL-C, high density lipoprotein cholesterol; LDL-C, low density lipoprotein cholesterol; Apo-A1, apolipoprotein A1; Apo-B, apolipoprotein B. Compared with HC, group, *p < 0.05. Compared with BKT, group, *p* < 0.05.

### 3.2 Serum metabolic profiling

**FIGURE 1:**
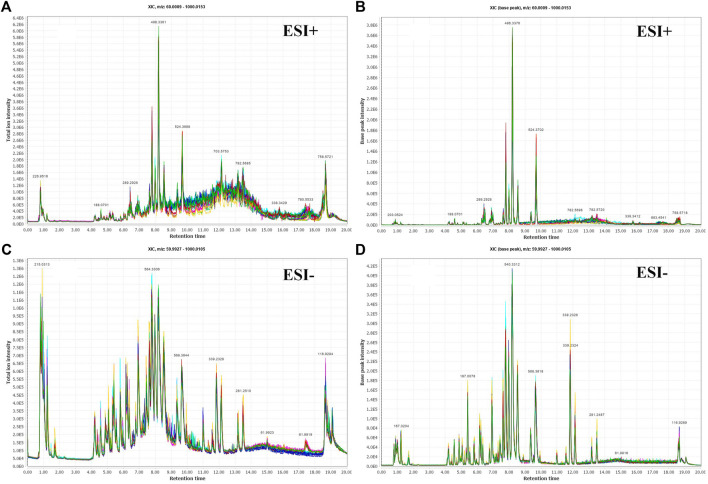
Serum fingerprint profiling of QC based on UPLC-MS/MS. Total ion chromatogram (TIC) and base peak intensity (BPI) diagram of ESI + model **(A,B)** and ESI- model **(C,D)**.

**FIGURE 2:**
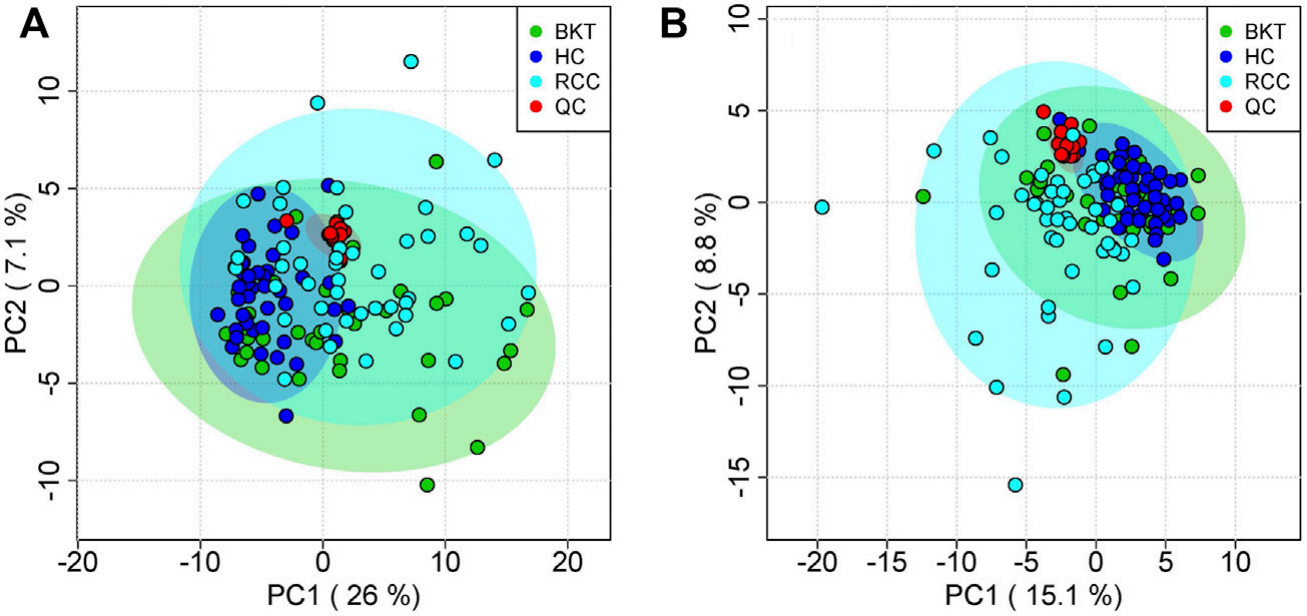
PCA analysis of QC samples and serum samples. PCA scores for QC samples and serum samples in ESI + mode **(A)** and ESI- mode **(B)**. HC, healthy control; BKT, benign kidney tumor; RCC, renal cell carcinoma; QC, quality control.

**FIGURE 3:**
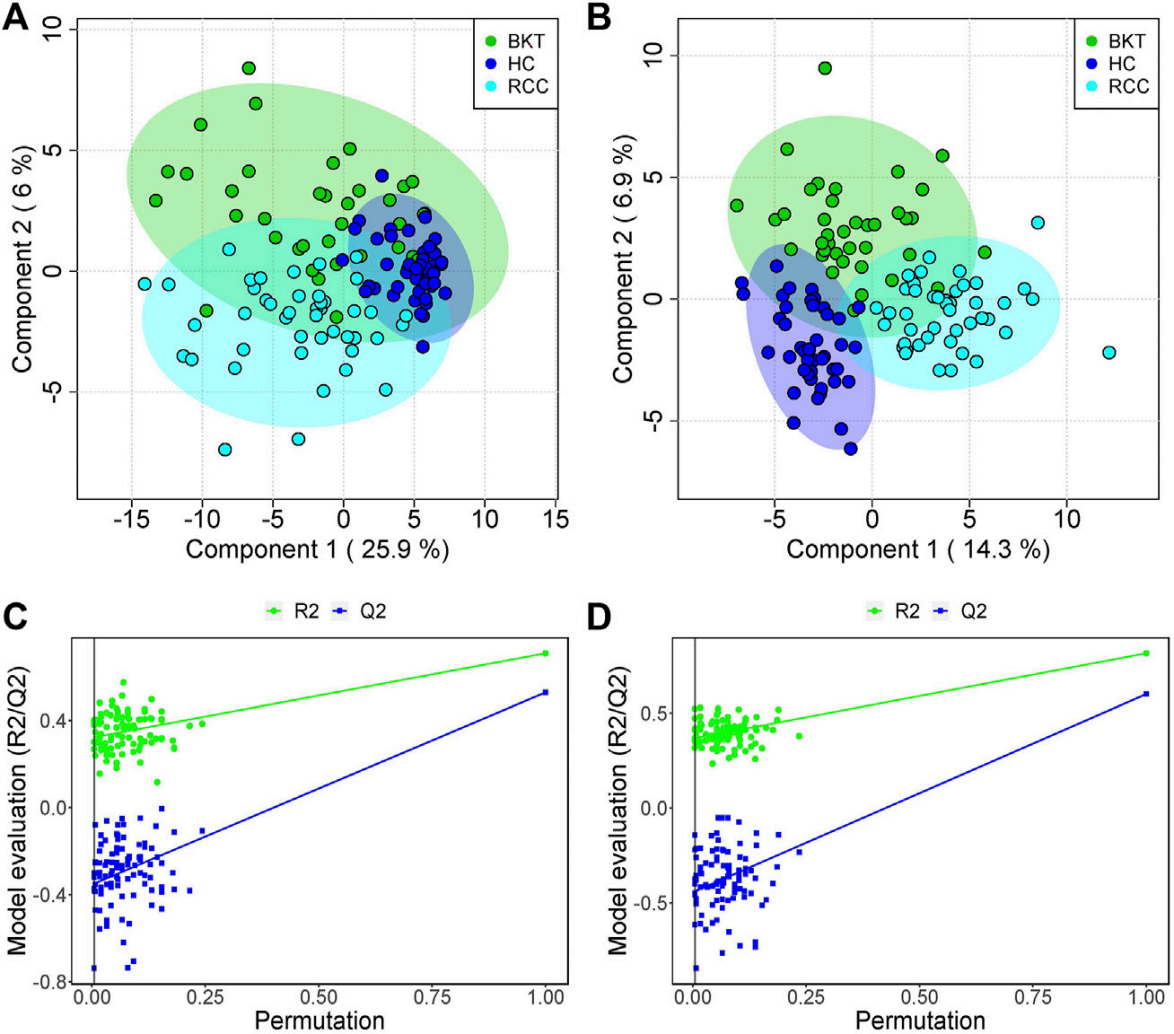
PLS-DA analysis and permutation test. PCA scores for the HC, BKT, and RCC groups in the ESI + model **(A)** and ESI- model **(B)** Permutation test in the ESI + model **(C)** and ESI- model **(D)** among HC group, BKT group, and RCC group. The criterion for PLS-DA not overfitting is that the R2 and Q2 values of all alignments on the left side are lower than the corresponding original points on the right side. The regression line of point Q2 intersects the horizontal coordinates or is less than 0. R2X and R2Y denote the degree of explanation of the PLS-DA model for the categorical variables X and Y, respectively. Q2 denotes the predictiveness of the PLS-DA model. HC, healthy control; BKT, benign kidney tumor; RCC, renal cell carcinoma.

The 131 serum samples and 14 QC samples were analyzed by UPLC-MS/MS in positive ion (ESI+) and negative ion (ESI-) modes. The results were analyzed by PCA analysis. The PCA score plots, total ion chromatogram (TIC) and base peak intensity (BPI) diagram of QC samples indicated that the metabolomics dataset of this study had good stability and reproducibility (Figure 1), while those of the serum samples showed that the serum components of the HC, BKT and RCC groups were not effectively separated (Figures 2A,B). Further analysis with PLS-DA resulted in effective separation (Figures 3A,B). To avoid overfitting of the PLS-DA model, 100 random permutation tests were performed. The results showed that for the HC, BKT and RCC groups, the degree of explanation of X (R2X), the degree of explanation of Y (R2Y) and the predictability of the model (Q2) were 0.32, 0.76, and 0.54, respectively, in the ESI + model, and were 0.37, 0.82 and 0.60, respectively, in the ESI- model (Figures 3C,D). These results reveals that the PLS-DA model has high goodness-of-fit and predictive power, and can effectively separate serum metabolite profiles of the HC, BKT, and RCC groups.

### 3.3 Screening of markers and analysis of their metabolic pathways

The results of MS/MS experiments were subjected to data filtering, which yielded 6938 and 6868 peaks in the ESI+ and ESI- modes, respectively. Qualitative analysis of these peaks using the standard compounds databases, customized databases, and integrated databases showed that a total of 910 metabolites were identified in ESI + mode and 917 metabolites were identified in ESI- mode. According to the criteria of variable importance for the projection>1, fold change>1.2 or<5/6, and *p* < 0.05 (Zhang et al., 2020b; Lu et al., 2021), 240 differential metabolites between the RCC and HC groups, 175 differential metabolites between the BKT and HC groups, and 64 differential metabolites between the BKT and RCC groups were identified.

**FIGURE 4:**
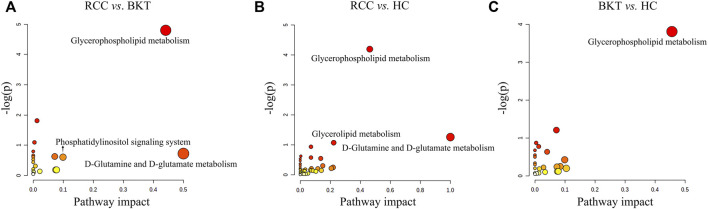
Metabolic pathway analysis of the screened differential markers. **(A)** Metabolic pathway analysis of differential metabolites between the RCC and BKT groups. **(B)** Metabolic pathway analysis of differential metabolites between the RCC and HC groups. **(C)** Metabolic pathway analysis of differential metabolites between the BKT and HC groups. HC, healthy control; BKT, benign kidney tumor; RCC, renal cell carcinoma; -log(P), negative logarithm of the *p*-value; Impact, impact value of metabolic pathway determined by topology analysis.

Pathway enrichment was used to analyze the metabolic pathways in which these differential metabolites may be involved. The results showed that among the 64 differential metabolites between the BKT and RCC groups, 8 differential metabolites were enriched in the glycerophospholipid metabolism pathway, 2 differential metabolites were enriched in the phosphatidylinositol signaling system, and 1 differential metabolite was enriched in the d-glutamine and d-glutamate metabolism pathway. Among the 240 differential metabolites between the RCC and HC groups, 10 differential metabolites were enriched in the glycerophospholipid metabolism pathway, 3 differential metabolites were enriched in the glycerolipid metabolism pathway, and 1 differential metabolite were enriched in the d-glutamine and d-glutamate metabolism pathway. These results indicate that the main metabolic pathways in patients with RCC may be lipid metabolism and amino acid metabolic pathways (Table 2 and Figure 4). Details of metabolic pathways matched to specific metabolites are shown in Supplementary Tables S2–S4.

**TABLE 2:** Metabolic pathways significantly altered between groups.

Group	Pathway	−log(*P*)	Impact	Hits	Compounds
RCC *vs.* BKT	Glycerophospholipid metabolism	11.10	0.44	8	PE 21:5, PC 34:2, LPC 19:2, PA 23:2, PS 18:3, PG 14:0, LPG 18:3 and PI 18:4
	Phosphatidylinositol signaling system	1.39	0.10	2	PI 18:4 and PA 23:2
	D-Glutamine and d-glutamate metabolism	1.67	0.50	1	Glutamic acid
RCC *vs.* HC	Glycerophospholipid metabolism	9.65	0.46	10	PE 21:5, PC (34:2), LPC 19:2, Choline, PA 23:2, PS 18:3, LPE 16:0, LPI 18:5, LPG 24:0 and PI 16:0
	Glycerolipid metabolism	8.48	0.22	3	PA 23:2, MG (18:1) and MGDG 22:3
	D-Glutamine and d-glutamate metabolism	2.90	1.00	1	Glutamic acid
BKT *vs.* HC	Glycerophospholipid metabolism	8.77	0.46	8	PE 22:1, PC 34:2, LPC 19:2, PA 23:3, PS 23:4, LPE 20:1, PG 14:0 and PI 16:0

Note: HC, healthy control; BKT, benign kidney tumor; RCC, renal cell carcinoma; PE, phosphatidylethanolamine; PC, phosphatidylcholine; LPC, lysophosphatidylcholine; PA, phosphatidic acid; PS, phosphatidylserine; LPE, lysophosphatidylethanolamine; LPI, lysophosphatidylinositol; LPG, lysophosphatidylglycerol; PI, phosphatidylinositol; MG, monoglyceride; MGDG, monogalactosyldiglyceride. -log(*P*), negative logarithm of the *p*-value of the statistic; Impact, impact value of metabolic pathway determined by topology analysis; Hits, the number of differential metabolites matching the pathway.

### 3.4 Diagnostic performance of the screened markers

**FIGURE 5:**
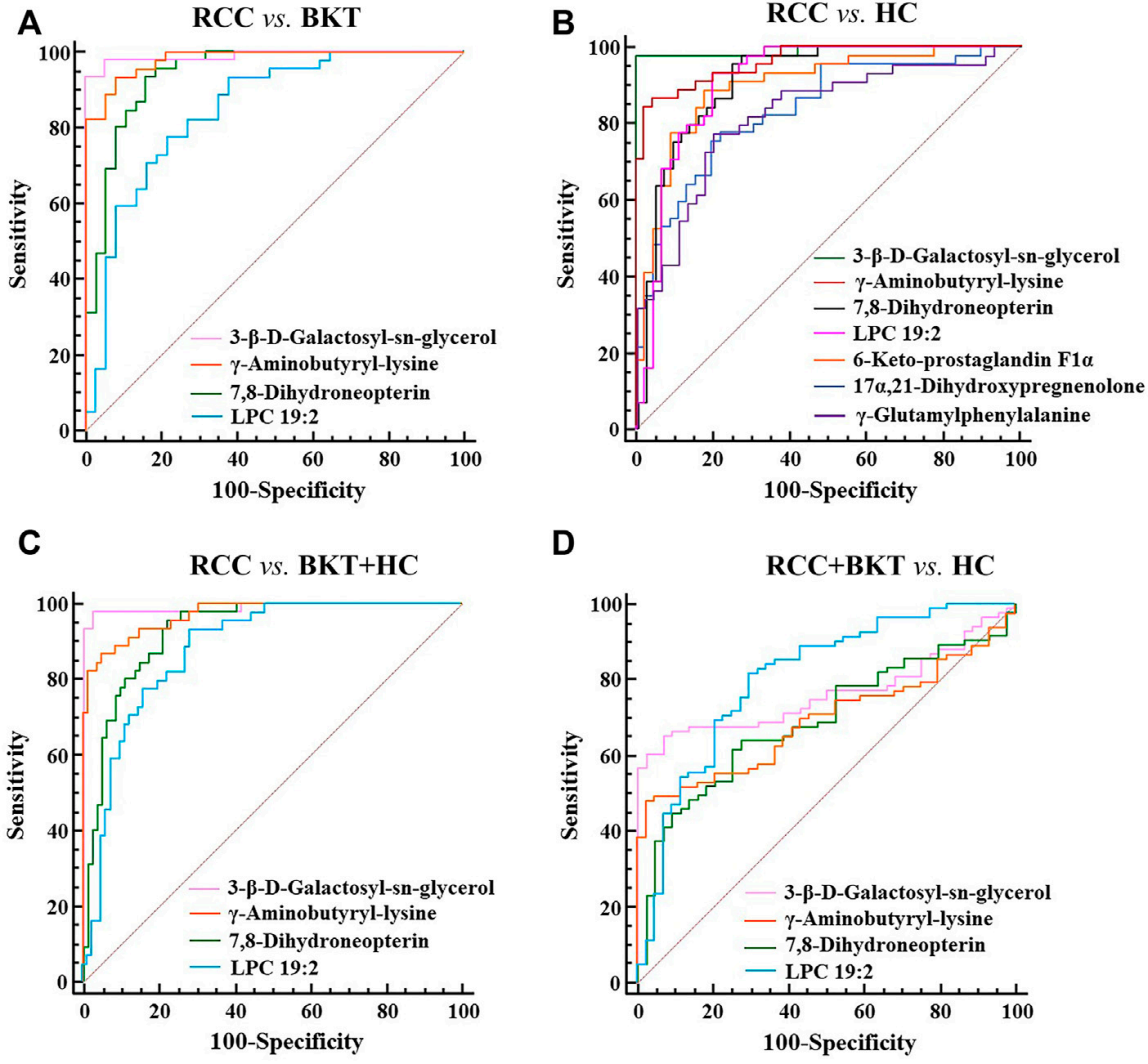
ROC analysis of candidate biomarkers. **(A)** ROC analysis of candidate biomarkers for discriminating RCC from BKT. **(B)** ROC analysis of candidate biomarkers for discriminating RCC from HC. **(C)** ROC analysis of candidate biomarkers for discriminating RCC from BKT and HC. **(D)** ROC analysis of candidate biomarkers for discriminating RCC and BKT from HC. HC, healthy control; BKT, benign kidney tumor; RCC, renal cell carcinoma. LPC, lysophosphatidylcholine.

**FIGURE 6:**
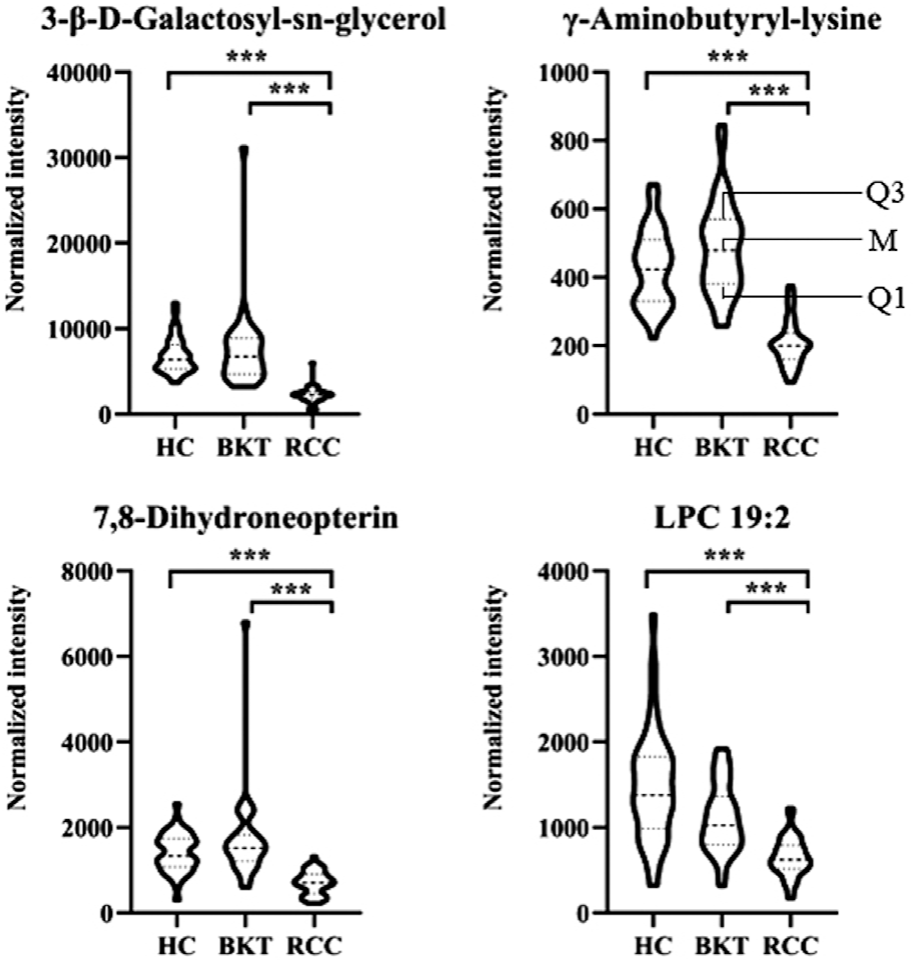
Normalized peak intensities of the four candidate biomarkers. HC, healthy control; BKT, benign kidney tumor; RCC, renal cell carcinoma. LPC, lysophosphatidylcholine. Normalization of peak intensities is performed using the MetNormalizer method of QC-based support vector regression analysis. QC, an aliquot mixture of all serum samples processed in the same way as the samples. Q1, 25th percentile; M, median; Q3, 75th percentile. ***p < 0.001.

With reference to the literature (Nahm, 2022), we selected differential metabolites with good or excellent diagnostic performance (AUC≥0.80) as potential markers for diagnosis. The diagnostic performance of the candidate markers was evaluated by ROC analysis, and the results showed that among the 64 differential metabolites between the BKT and RCC groups, there were 4 differential metabolites with area under the ROC curve (AUC)≥0.80, namely 3-β-D-Galactosyl-sn-glycerol, γ-Aminobutyryl-lysine, 7,8-Dihydroneopterin, and lysophosphatidylcholine (LPC) 19:2 (Table 3). Among the 240 differential metabolites between the HC and RCC groups, there were 7 differential metabolites with AUC≥0.80, namely 3-β-D-Galactosyl-sn-glycerol, γ-Aminobutyryl-lysine, 7,8-Dihydroneopterin, LPC 19:2, 6 Keto-prostaglandin F1α, 17α,21-Dihydroxypregnenolone, and γ-Glutamylphenylalanine (Figure 5). The m/z of parent ion and product ion of the 7 differential metabolites is shown in Supplementary Table S5. Serum levels of 6-Keto-prostaglandin F1α, 17α,21-Dihydroxypregnenolone and γ-Glutamylphenylalanine were significantly higher in the RCC group compared with the HC group (Table 4). Compared with the HC and BKT groups, serum levels of 3-β-D-Galactosyl-sn-glycerol, γ-Aminobutyryl-lysine, 7,8-Dihydroneopterin and LPC 19:2 were significantly lower in the RCC group, and the normalized peak intensities of these four metabolites were shown in Figure 6. These results suggest that 3-β-D-Galactosyl-sn-glycerol, γ-Aminobutyryl-lysine, 7,8-Dihydroneopterin and LPC 19:2 could distinguish patients with RCC from patients with BKT and healthy subjects.

**TABLE 3:** Diagnostic performance evaluation of candidate biomarkers.

Biomarker	Scan mode	Rt (s)	m/z	Adducts	AUC (95%CI)	Se (%)	Sp (%)	YI
RCC vs. BKT								
3-β-D-Galactosyl-sn-glycerol	—	—	—	—	0.989 (0.936–1.000)	93.33	100.00	0.933
γ-Aminobutyryl-lysine	—	—	—	—	0.981 (0.924–0.999)	93.33	92.11	0.854
7,8-Dihydroneopterin	—	—	—	—	0.941 (0.867–0.981)	93.33	84.21	0.775
LPC 19:2	—	—	—	—	0.845 (0.748–0.916)	77.27	78.38	0.557
RCC vs. HC								
3-β-D-Galactosyl-sn-glycerol	ESI-	481.035	253.083	M-H	0.990 (0.942–1.000)	97.73	100.00	0.977
γ-Aminobutyryl-lysine	ESI-	545.151	230.155	M-H	0.962 (0.898–0.991)	86.36	95.56	0.819
7,8-Dihydroneopterin	ESI-	481.173	254.086	M-H	0.916 (0.838–0.964)	97.73	73.33	0.711
LPC 19:2	ESI+	440.903	556.346	M + Na	0.909 (0.829–0.959)	93.18	80.00	0.732
6-Keto-prostaglandin F1α	ESI+	668.909	371.242	M + H	0.897 (0.815–0.952)	88.64	82.22	0.709
17α,21-Dihydroxypregnenolone	ESI+	698.951	349.237	M + H	0.830 (0.735–0.901)	77.27	77.78	0.551
γ-Glutamylphenylalanine	ESI-	283.958	293.113	M-H	0.823 (0.728–0.896)	77.27	80.00	0.573
RCC vs (BKT + HC)								
3-β-D-Galactosyl-sn-glycerol	—	—	—	—	0.990 (0.953–0.999)	97.78	97.56	0.953
γ-Aminobutyryl-lysine	—	—	—	—	0.971 (0.924–0.992)	86.67	95.12	0.818
7,8-Dihydroneopterin	—	—	—	—	0.928 (0.868–0.966)	95.56	78.05	0.736
LPC 19:2	—	—	—	—	0.879 (0.808–0.930)	93.18	71.60	0.648
(RCC + BKT) vs HC								
3-β-D-Galactosyl-sn-glycerol	—	—	—	—	0.763 (0.679–0.834)	65.06	93.18	0.582
γ-Aminobutyryl-lysine	—	—	—	—	0.692 (0.604–0.771)	48.19	97.73	0.459
7,8-Dihydroneopterin	—	—	—	—	0.691 (0.602–0.770)	63.86	72.73	0.366
LPC 19:2	—	—	—	—	0.803 (0.722–0.869)	81.48	70.45	0.519

Note: HC, healthy control; BKT, benign kidney tumor; RCC, renal cell carcinoma; Rt, retention time; m/z, mass-to-charge ratio; LPC, lysophosphaticholine; AUC, area under ROC, curve; 95%CI, 95%confidence interval; Se, sensitivity; Sp, specificity; YI, youden index.

**TABLE 4:** Significantly differential metabolites of RCC vs. BKT groups and RCC vs. HC groups.

Group	FC	VIP	*P*	Trend	Pathway
RCC vs. BKT					
3-β-D-Galactosyl-sn-glycerol	0.296	3.723	<0.001	↓	Galactose metabolism
γ-Aminobutyryl-lysine	0.425	4.769	<0.001	↓	Amino acid metabolism
7,8-Dihydroneopterin	0.419	3.349	<0.001	↓	Folate biosynthesis
LPC 19:2	0.589	4.611	<0.001	↓	Glycerophospholipid metabolism
RCC vs. HC					
3-β-D-Galactosyl-sn-glycerol	0.322	3.853	<0.001	↓	Galactose metabolism
γ-Aminobutyryl-lysine	0.485	3.687	<0.001	↓	Amino acid metabolism
7,8-Dihydroneopterin	0.500	3.167	<0.001	↓	Folate biosynthesis
LPC 19:2	0.441	3.239	<0.001	↓	Glycerophospholipid metabolism
6-Keto-prostaglandin F1α	2.800	3.042	<0.001	↑	Arachidonic Acid Metabolism
17α,21-Dihydroxypregnenolone	2.119	2.722	<0.001	↑	Steroid hormone biosynthesis
γ-Glutamylphenylalanine	1.932	1.659	0.006	↑	Amino acid metabolism

Note: HC, healthy control; BKT, benign kidney tumor; RCC, renal cell carcinoma; LPC, lysophosphatidylcholine; FC, fold change; VIP, variable importance for the projection.

### 3.5 Correlation analysis of markers with common kidney function and lipid indicators

Spearman correlation was used to analyze the correlation of the four metabolites with common kidney function and lipid indicators (Table 5). The results showed that 3-β-D-Galactosyl-sn-glycerol was negatively correlated with Cr (r = -0.268, *p* = 0.002), CysC (r = -0.268, *p* = 0.002) and NCAL (r = -0.176, *p* = 0.047), while positively correlated with eGFR (r = 0.268, *p* = 0.002). 7,8-Dihydroneopterin was negatively correlated with Cr (r = -0.214, *p* = 0.016), CysC (r = -0.193, *p* = 0.030) and NGAL (r = -0.202, *p* = 0.022), whereas positively correlated with eGFR (r = 0.193, *p* = 0.030). LPC 19:2 had negative correlation with urea (r = -0.181, *p* = 0.042), Cr (r = -0.222, *p* = 0.012), CysC (r = -0.202, *p* = 0.023) and TG (r = -0.183 *p* = 0.040), but positively correlation with eGFR (r = 0.202, *p* = 0.023). Although the correlation coefficient is weak (Akoglu, 2018; Schober et al., 2018), these results indicated that the reduced serum levels of 3-β-D-Galactosyl-sn-glycerol, 7,8-Dihydroneopterin and LPC 19:2 in patients with RCC may have a certain degree of correlation with kidney impairment and dyslipidemia.

**TABLE 5:** Correlation analysis of markers with common renal function and lipid indicators (r, *P*).

Compounds	3-β-D-Galactosyl-sn-glycerol	γ-Aminobutyryl-lysine	7,8-Dihydroneopterin	LPC 19:2
Urea (mmol/L)	−0.159, 0.093	−0.029, 0.748	0.037, 0.680	−0.181, 0.042*
Cr (μmol/L)	−0.268, 0.002*	−0.095, 0.290	−0.214, 0.016*	−0.222, 0.012*
UA (μmol/L)	−0.084, 0.350	−0.074, 0.407	−0.002, 0.986	−0.041, 0.650
CysC (mg/L)	−0.271, 0.002*	−0.138, 0.123	−0.190, 0.033*	−0.203, 0.023*
eGFR (ml/min/1.73m^2^)	0.271, 0.002*	0.138, 0.123	0.190, 0.033*	0.203, 0.023*
C1q (mg/L)	0.081, 0.367	−0.015, 0.869	0.006, 0.949	0.029, 0.746
NGAL (μg/L)	−0.169, 0.063	−0.131, 0.152	−0.218, 0.016*	−0.125, 0.173
TC (mmol/L)	−0.057, 0.523	−0.054, 0.547	−0.015, 0.169	−0.101, 0.263
TG (mmol/L)	−0.113, 0.204	−0.132, 0.138	−0.169, 0.057	−0.176, 0.049*
HDL (mmol/L)	0.084, 0.345	0.102, 0.255	0.104, 0.243	0.115, 0.203
LDL (mmol/L)	−0.053, 0.555	−0.044, 0.626	0.044, 0.620	−0.150, 0.094
APO-A (g/L)	0.101, 0.262	0.102, 0.255	−0.063, 0.486	0.123, 0.173
APO-B (g/L)	−0.071, 0.427	−0.076, 0.395	−0.007, 0.939	−0.168, 0.062

Note: LPC, lysophosphatidylcholine; Cr, creatinine; UA, uric acid; CysC, cystatin C; C1q, complement C1q; NGAL, neutrophil gelatinase-associated lipocalin; eGFR, estimated glomerular filtration rate; TC, total cholesterol; TG, triglyceride; HDL-C, high density lipoprotein cholesterol; LDL-C, low density lipoprotein cholesterol; Apo-A1, apolipoprotein A1; Apo-B, apolipoprotein B. *p < 0.05.

## 4 Discussion

RCC is a metabolic disease and analyzing its metabolic profile is essential for identification of biomarkers. Metabolomics based on UPLC-MS/MS technology have been widely used to explore new biomarkers of diseases (Liu et al., 2019b; Crestani et al., 2020; Wilkinson et al., 2020; Ai et al., 2022). Studies have shown that comparing with plasma samples. Serum can avoid the influence of anticoagulants on the concentration of certain metabolites (such as amino acids) compared to plasma samples, and the higher concentration of metabolites in serum may provide more sensitive results in biomarker detection, so the metabolomic analysis of serum samples may be more reliable than plasma samples (Yu et al., 2011; Sotelo-Orozco et al., 2021). Take into account this factor, serum was selected as the sample for this study. We found that there were significant differences in serum metabolic profiles among healthy subjects, patients with BKT, and patients with RCC. The glycerophospholipid metabolism pathway, d-glutamine and d-glutamate metabolism pathway, phosphatidylinositol signaling system, glycerolipid metabolism pathway, galactose metabolism pathway, and folate biosynthesis were significantly abnormal in patients with RCC. In addition, 3-β-D-Galactosyl-sn-glycerol, γ-Aminobutyryl-lysine, 7,8-Dihydroneopterin, and LPC 19:2 were identified as potential markers for the diagnosis of RCC.

Dysregulated lipid metabolism affects a variety of cellular physiological processes, such as cell proliferation, differentiation and motility, which are closely associated with cancer transformation, progression and metastasis (Perrotti et al., 2016; Butler et al., 2020). In the present study, most of the markers screened from RCC were lipid and lipid-like metabolites, among which serum levels of 3-β-D-Galactosyl-sn-glycerol and LPC 19:2 were significantly reduced, while serum levels of 6-Keto-prostaglandin F1α and 17α,21-Dihydroxypregnenolone were significantly increased. The reduced serum levels of lipid and lipid-like metabolites in patients with RCC may be attributed to increased demand for phospholipids by the rapidly proliferated cancer cells to generate new cell membranes; and, the increased serum levels of lipid and lipid-like metabolites may because the enhanced exogenous lipid uptake and activated endogenous lipid synthesis in tumor cells to provide energy (Butler et al., 2020). It is worth noticing that LPC 19:2 in serum has not been reported in the literature. In this study, the retention time, MS1 and MS2 data obtained through MS/MS analysis matched with the integrated database, and LPC 19:2 was identified. After metabolic pathway analysis, it was found that LPC 19:2 involved in the glycerol phospholipid metabolic pathway. Glycerophospholipid metabolism is one of the important pathways of lipid metabolism *in vivo*, and changes in glycerophospholipid levels may affect cellular function, cytocytosis, cytospin, cytoskeletal regulation, and membrane fusion (Wang et al., 2021b). Previous studies have found that there is dysregulated glycerophospholipid metabolism pathway in some cancers, such as gastric cancer (Yu et al., 2021), hepatocellular carcinoma (Yu et al., 2022), prostate cancer (Xu et al., 2021), non-small cell lung cancer (Chen et al., 2018), ovarian cancer (Gan et al., 2020), colorectal cancer (Gumpenberger et al., 2021), and pancreatic ductal adenocarcinoma (Martin-Blazquez et al., 2020). In this study, we found altered glycerophospholipid metabolism pathway in patients with RCC, further confirming that the glycerophospholipid metabolism pathway may be associated with the occurrence of RCC. However, the mechanism underlying the altered the glycerophospholipid metabolic pathway in RCC needs further study. In addition, abnormal lipid metabolism in RCC may also be due to abnormal expression of key genes of lipogenesis, such as fatty acid synthase, ATP citrate lyase, sterol regulatory element-binding protein 1, and hydroxy acyl-CoA dehydrogenase alpha subunit (Heravi et al., 2022). Further studies at gene level are needed to understand the mechanisms.

Glutamine metabolism and glutamic acid metabolism are one of the important pathways to obtain nutrients during the growth and proliferation of cancer cells. Glutamine is not only the nitrogen source for cancer cell biosynthesis (such as nucleotide synthesis and protein synthesis), either directly or indirectly by conversion to glutamic acid, but also a carbon source for amino acid and fatty acid synthesis in cancer cells (Altman et al., 2016). Additionally, it can be converted to α-ketoglutarate to enter the tricarboxylic acid cycle, thus providing energy for cancer cell growth and proliferation (Altman et al., 2016). One study found (Shroff et al., 2015) that the metabolites of the glutaminolytic pathway were elevated in RCC tissues compared to normal kidney tissues, suggesting that RCC survival may be related with glutamine metabolism. Consistently, the current study confirmed that serum levels of glutamic acid, which is involved in the d-glutamine and d-glutamate metabolism pathway, were elevated in patients with RCC. These findings indicate that elevated glutamic acid in RCC may provide sufficient energy and substances for growth and proliferation of RCC.

In this study, 3-β-D-Galactosyl-sn-glycerol, γ-Aminobutyryl-lysine and 7,8-Dihydroneopterin were identified to be possible potential biomarkers to distinguish patients with RCC from patients with BKT and healthy subjects. The chosen kidney function and lipid indicators for comparative analysis in this study are commonly used in clinical diagnosis of kidney function damage or to judge whether the blood lipid level is normal. They are intermediate or end products of substance metabolism in the body. Because of their high level in blood, they can be accurately detected by conventional chemical analysis methods. In contrast, the new biomarkers screened in this study were obtained by untargeted metabolomics technology, which can only perform qualitative and semi-quantitative analysis on the analyzed substances (that is, the level of substance in the sample can only be estimated roughly according to the peak height or area of the mass spectrum). However, several potential biomarkers for diagnosis or differential diagnosis of RCC, which were not previously noticed, were found through the analysis of serum samples of subjects using untargeted metabolomics technology in this study. Many studies have also confirmed that untargeted metabolomics can comprehensively and systematically analyze small molecular substances in biological samples (Hou et al., 2020; Castelli et al., 2022; Khoramipour et al., 2022), and it is an excellent analytical tool for screening biomarkers at present. In this group of subjects, no indicators consistent with the observed kidney function and blood lipid indicators were found by UPLC-MS/MS method. This is because we use untargeted metabolomics technology to screen different markers in different settings. However, the observed kidney function and blood lipid indicators have high concentrations in each group we set, so they may be ignored as non-differential indicators during the analysis. Some new biomarkers related to them were also found. However, due to different detection methods, the correlation between these new biomarkers and any of the observed kidney function and blood lipid indicators is not good enough. However, this is enough to show that the reduced serum levels of 3-β-D-Galactosyl-sn-glycerol and 7,8-Dihydroneopterin in patients with RCC may have a certain of correlation with kidney impairment and dyslipidemia. However, the underlying mechanisms need further investigation. 3-β-D-Galactosyl-sn-glycerol is involved in the galactose metabolism pathway and it is reduced in the serum of patients with RCC. Lactose is hydrolyzed into to glucose and galactose in the intestine by lactase (Forsgard, 2019), which can enter the circulation through the same transporter in the intestinal epithelium and participate in metabolism. The glucose requirements of cancer cells are greater than those of normal cells because they mainly depend on aerobic glycolysis for their energy source. Therefore, reduced serum levels of 3-β-D-Galactosyl-sn-glycerol metabolites involved in galactose metabolism in patients with RCC may be attributed to the competitive inhibition of galactose uptake in intestinal epithelial cells. Although the 7,8-dihydroneopterin in other diseases has been reported, the mechanism of its association with RCC occurrence needs to be further investigated. 7,8-Dihydroneopterins are organic compounds of biopterins and their derivatives, which have antioxidant effects (Janmale et al., 2020). The current study identified γ-Aminobutyryl-lysine, a dipeptide present in the human brain, whose relationship with RCC has not been reported.

In this study, serum levels of 6-Keto-prostaglandin F1α, 17α,21-Dihydroxypregnenolone and γ-Glutamylphenylalanine in RCC patients were found to be elevated which may be potential biomarkers to distinguish RCC patients from healthy individuals. However, the diagnostic performances of these three biomarkers for distinguishing RCC and BKT were not good (all AUC<0.80, the data were not displayed). 6-Keto-prostaglandin F1α belongs to the prostaglandin and related compounds. Prostaglandin is unsaturated fatty acids produced by the cyclooxygenase-catalyzed arachidonic acid metabolism and is widely present in various vital tissues and body fluids in humans. It is reported that cyclooxygenase-2 overexpression in RCC may be associated with metastasis of tumor cells, tumor invasion and angiogenesis (Kaminska et al., 2014; Ching et al., 2020). Therefore, elevated 6-Keto-prostaglandin F1α in RCC patients may be attributed to the overexpression of cyclooxygenase. 17α,21-Dihydroxypregnenolone belongs to the organic compounds known as 21-hydroxysteroids, and is involved in steroid hormone biosynthesis pathway. Increased serum levels of 17α,21-Dihydroxypregnenolone in patients with RCC may be related to the expression and function of steroid hormone receptors. These receptors can act as ligand-dependent transcription factors or induce gene expression through ligand-independent pathways and play an important role in tumor growth and differentiation (Bennett et al., 2014). γ-Glutamylphenylalanine is a dipeptide composed of γ-glutamic acid and phenylalanine, which is a proteolytic breakdown product of larger proteins. It may be formed by γ-glutamyl transpeptidase catalyzing the transpeptidation between glutathione and the corresponding amino acid. γ-glutamyl transpeptidase is an enzyme primarily involved in cellular glutathione homeostasis, and although it has been studied in other cancers, such as gastric cancer (Wang et al., 2017), intrahepatic cholangiocarcinoma (Zhang et al., 2021), hepatocellular carcinoma (Ince et al., 2020) and oral squamous cell carcinoma (Mujawar et al., 2020), its mechanism of action in RCC has not been reported. In addition, some compounds have not been screened as potential diagnostic biomarkers of RCC in this study, such as 21 Deoxycortisol, Dehydrocholic acid, Leucyl leucine, Ethylene brassylate, Leu Ala OH and Leukotriene F4, etc. However, they also show high FC, VIP scores and significant *p*-values. Therefore, these compounds may also be worthy of attention and further research.

In the screening process of biomarkers, it was found that some compounds involved two or more metabolic pathways, such as phosphatidic acid (PA) 23:2 and phosphatidylinositol (PI) 18:4, and were associated with glycerophospholipid, phosphatidylinositol and glycerolipid pathways. Since these biomarkers are provided by database comparison, are they the intersections of metabolic pathways or subset of other pathways? Unclear. Fortunately, these substances have little significance for this study, so they are only cared about very little. Although these metabolites are not as important as those screened in this study, they actually involve some metabolic pathways. Perhaps these metabolites could be related to a certain disease in a particular way, but they cannot be observed in this study. Similarly, in the process of pathway analysis, it is found that some metabolic pathways only involve the changes of 1-2 markers, for example, only PA 23:2 and PI 18:4 were hit to the phosphatidylinositol metabolism pathway and only glutamic acid was hit to the d-glutamine and d-glutamate metabolism pathway. Although these substances were discovered by screening, the final results only focused on 4 biomarkers, indicating that the changes of 1-2 compounds may not affect the changes of the whole metabolic pathway. In addition, the changes in their quantity (peak intensity and area of MS) of these substances are not large enough among the different study groups. Therefore, they were not finally screened as differential biomarkers.

Based on the above theories and research findings, we propose a hypothesis: there are some changes in metabolic pathways of substances in the body of RCC patients, which at least include lipid metabolism, amino acid metabolism, galactose metabolism and folate biosynthesis. Studying the signal pathways involved in these metabolic pathways may find a new way to explore the pathogenesis of RCC. This study only provides a new way for screening biomarkers for diagnosis and differential diagnosis of RCC. In the follow-up study, we will verify and evaluate the diagnostic performance of these potential biomarkers of RCC found in this study through targeted metabolomic analysis technology based on the research results, so as to explore the possibility of these biomarkers in clinical practice.

## 5 Conclusion

In summary, this study analyzed serum metabolic profiles of healthy subjects, patients with BKT and patients with RCC using UPLC-MS/MS. Four potential biomarkers for the diagnosis of RCC were screened by multivariate statistical analysis and ROC analysis, namely LPC 19:2 involved in the glycerophospholipid metabolism pathway, 3-β-D-Galactosyl-sn-glycerol involved in the galactose metabolism, 7,8-Dihydroneopterin involved in the folate biosynthesis and γ-Aminobutyryl-lysine, an amino acid metabolite. In addition to the above metabolic pathways, there may also be changes in the phosphatidylinositol signaling system, the d-glutamine and d-glutamate metabolism pathway, and the glycerolipid metabolism pathway in patients with RCC. These results suggest that the occurrence of RCC may be associated with changes in lipid metabolism, amino acid metabolism, galactose metabolism, and folate biosynthesis. These four metabolites may become markers for the diagnosis or differential diagnosis of RCC. However, this study is limited in the small sample size and the lack of targeted metabolomics validation. Therefore, follow-up studies with larger sample size and validation with other omics such as genomics and proteomics are warranted to clarify the metabolic mechanism of RCC and to further identify tumor markers for clinical application.

## Data availabilty statement

The original contributions presented in the study are included in the article/Supplementary Materials, further inquiries can be directed to the corresponding author.
