# Supplementary material for: Study on potential markers for diagnosis of renal cell carcinoma by serum untargeted metabolomics based on UPLC-MS/MS
Source: Front Physiol. 2022 Nov 29;13:996248. doi: 10.3389/fphys.2022.996248 (PMC9745078; doi:10.3389/fphys.2022.996248)
Supplement: Supplementary file 1 [file Table1.docx]

**Supplementary Table 1. Demographic characteristics of the study subjects.**

| **Group** | **HC (n=45)** | **BKT (n=40)** | **RCC (n=46)** | **χ^2^/F, *P*** |
| --- | --- | --- | --- | --- |
| Male/female (n) | 23/22 | 27/13 | 34/12 | 5.426^a^,0.066 |
| Age (year) | 49.6 ± 11.3 | 49.7 ± 14.2 | 52.7 ± 11.4 | 0.928^b^, 0.398 |
| Tumor histological subtype (n) |  |  |  |  |
| Clear Cell | － | － | 35 | － |
| Papillary | － | － | 3 | － |
| Chromophobe | － | － | 1 | － |
| Unclassified | － | － | 7 | － |
| Fuhrman grade (n) |  |  |  |  |
| Ⅰ | － | － | 19 | － |
| Ⅱ | － | － | 12 | － |
| Ⅲ | － | － | 8 | － |
| Ⅳ | － | － | 7 | － |
| TNM stage (n) |  |  |  |  |
| Ⅰ | － | － | 9 | － |
| Ⅱ | － | － | 4 | － |
| Ⅲ | － | － | 7 | － |
| Ⅳ | － | － | 6 | － |
| Unknown | － | － | 20 | － |

Note: n, case; a is the χ2 value; b is the value of F; HC, healthy control; BKT, benign kidney tumor; RCC, renal cell carcinoma.

**Supplementary Table 2. Identification data of metabolites with significant differences between the RCC and BKT groups and the metabolic pathways involved.**

| **varID** | **Scan mode** | **Compounds** | **FC** | ***P*** | **VIP** | **m/z** | **Rt(s)** | **Adducts** | **pathway** |
| --- | --- | --- | --- | --- | --- | --- | --- | --- | --- |
| V1 | ESI+ | (3R)-β-Leucine | 1.245 | 0.001 | 2.888 | 132.102 | 125.604 | M+H | NA |
| V2 |  | Glutamic acid | 1.278 | 0.025 | 1.986 | 148.061 | 54.102 | M+H | D-Glutamine and D-glutamate metabolism |
| V3 |  | Cis-5-Dodecenoic acid | 0.824 | ＜0.001 | 3.235 | 199.169 | 647.008 | M+H | NA |
| V4 |  | L-Acetylcarnitine | 1.203 | 0.048 | 1.724 | 204.122 | 57.868 | M+H | NA |
| V5 |  | 4-oxo 2-Nonenal-d3 | 1.291 | 0.026 | 1.887 | 158.118 | 281.869 | M+H | NA |
| V6 |  | α-Amino-γ-cyanobutanoate | 1.568 | 0.003 | 2.480 | 129.066 | 56.965 | M+H | NA |
| V7 |  | Indolelactic acid | 1.275 | 0.032 | 1.791 | 206.081 | 328.871 | M+H | NA |
| V8 |  | (±)-Hexanoylcarnitine | 1.363 | 0.013 | 2.105 | 260.177 | 311.444 | M+H | NA |
| V9 |  | γ-Glu-Leu | 1.337 | 0.021 | 2.069 | 261.144 | 277.433 | M+H | NA |
| V10 |  | O-Phospho-L-Tyrosine | 1.276 | 0.013 | 2.113 | 262.038 | 115.047 | M+H | NA |
| V11 |  | N-(1-Deoxy-1-fructosyl)valine | 1.310 | 0.009 | 2.188 | 280.139 | 74.198 | M+H | NA |
| V12 |  | All-cis-4,7,10,13,16-docosapentaenoic acid | 1.380 | 0.015 | 2.078 | 331.265 | 727.270 | M+H | NA |
| V13 |  | Docosahexaenoic Acid-d5 | 0.804 | 0.034 | 1.877 | 334.274 | 712.376 | M+H | NA |
| V14 |  | N-2-[4-(3,3-Dimethylallyloxy)phenyl]ethylcinnamide | 1.381 | 0.039 | 1.743 | 336.191 | 268.740 | M+H | NA |
| V15 |  | 1-Monopalmitin | 0.796 | 0.019 | 2.011 | 353.268 | 717.454 | M+Na | NA |
| V16 |  | Calcitriol | 1.208 | 0.022 | 1.959 | 399.326 | 667.603 | M+H-H2O | Steroid biosynthesis |
| V17 |  | N6-Carbamoyl-L-threonyladenosine | 1.264 | 0.002 | 2.555 | 413.142 | 272.314 | M+H | NA |
| V18 |  | 23-Acetoxysoladulcidine | 1.279 | 0.047 | 1.670 | 474.367 | 572.560 | M+H | NA |
| V19 |  | LPC 20:4 | 0.812 | 0.037 | 1.823 | 544.349 | 581.195 | M+H | Glycerophospholipid metabolism |
| V20 |  | LPC 19:2 | 0.589 | ＜0.001 | 4.611 | 556.347 | 440.903 | M+Na | Glycerophospholipid metabolism |
| V21 |  | SM d28:2 | 0.798 | 0.002 | 2.737 | 617.468 | 469.542 | M+H | NA |
| V22 |  | Cer-AP t39:3 | 0.733 | 0.029 | 2.019 | 636.556 | 1001.108 | M+H | NA |
| V23 |  | PC(O-34:2) | 0.683 | 0.004 | 2.603 | 744.590 | 1119.840 | M+H | NA |
| V24 |  | PC 34:2 | 1.321 | 0.017 | 1.986 | 758.570 | 659.887 | M+H | Glycerophospholipid metabolism; Linoleic acid metabolism |
| V25 |  | PC 38:4 | 0.823 | 0.010 | 2.269 | 810.601 | 712.505 | M+H | Glycerophospholipid metabolism; Linoleic acid metabolism |
| V26 |  | 4α-hydroxymethyl-5α-cholesta-8,24-dien-3β-ol | 1.378 | 0.020 | 2.012 | 415.357 | 941.116 | M+H | NA |
| V27 | ESI- | Carbamate | 1.243 | 0.014 | 1.471 | 60.000 | 811.847 | M-H | NA |
| V28 |  | 2-Hydroxy-3-methylbutyric acid | 1.360 | 0.003 | 1.807 | 117.057 | 277.438 | M-H | NA |
| V29 |  | 6-Hydroxyhexanoic acid | 1.298 | 0.022 | 1.383 | 131.072 | 312.002 | M-H | NA |
| V30 |  | Dl-P-Hydroxyphenyl lactic acid | 1.401 | ＜0.001 | 2.169 | 181.051 | 281.198 | M-H | NA |
| V31 |  | Sulfosalicylic acid | 1.369 | 0.017 | 1.381 | 216.981 | 277.215 | M-H | NA |
| V32 |  | Pantothenic acid | 1.231 | 0.008 | 1.652 | 218.103 | 263.418 | M-H | Pantothenate and CoA biosynthesis |
| V33 |  | 5-Acetylamino-6-formylamino-3-methyluracil | 1.395 | 0.014 | 1.569 | 225.069 | 1117.890 | M-H | Caffeine metabolism |
| V34 |  | γ-Aminobutyryl-lysine | 0.425 | ＜0.001 | 4.769 | 230.155 | 545.151 | M-H | NA |
| V35 |  | Curcumenone | 0.530 | ＜0.001 | 4.878 | 233.155 | 523.757 | M-H | NA |
| V36 |  | Isoleucyl-Hydroxyproline | 1.671 | 0.002 | 1.736 | 243.135 | 307.117 | M-H | NA |
| V37 |  | Aspartyl-Asparagine | 1.295 | 0.034 | 1.471 | 246.075 | 1117.900 | M-H | NA |
| V38 |  | 3-β-D-Galactosyl-sn-glycerol | 0.296 | ＜0.001 | 3.723 | 253.083 | 481.035 | M-H | Galactose metabolism |
| V39 |  | 7,8-Dihydroneopterin | 0.419 | ＜0.001 | 3.349 | 254.086 | 481.173 | M-H | Folate biosynthesis |
| V40 |  | Peptide 2-(3-carboxy-3-aminopropyl)-L-histidine | 1.348 | 0.011 | 1.466 | 255.114 | 342.795 | M-H | NA |
| V41 |  | 8-Oxohexadecanoic acid | 1.200 | 0.027 | 1.304 | 269.213 | 791.868 | M-H | NA |
| V42 |  | Formylmethionyl-leucyl-phenylalanine methyl ester | 1.506 | 0.010 | 1.517 | 277.155 | 299.793 | M-H | NA |
| V43 |  | D-Norvaline | 0.725 | 0.049 | 1.467 | 293.175 | 420.113 | 2M+Hac-H | NA |
| V44 |  | N-Acetylneuraminate | 1.362 | 0.016 | 1.650 | 309.108 | 257.631 | M-H | Amino sugar and nucleotide sugar metabolism |
| V45 |  | Lansamide 4 | 1.310 | 0.001 | 1.960 | 310.151 | 107.224 | M-H | NA |
| V46 |  | FA 22:4 | 1.207 | 0.027 | 1.420 | 331.264 | 795.492 | M-H | NA |
| V47 |  | Melibiitol | 1.346 | 0.003 | 1.792 | 343.128 | 276.314 | M-H | Galactose metabolism; Phosphatidylinositol signaling system |
| V48 |  | N-(Carbethoxyacetyl)-4-chloro-L-tryptophan | 1.225 | 0.042 | 1.771 | 351.078 | 107.592 | M-H | NA |
| V49 |  | Trimethobenzamide | 1.408 | 0.016 | 1.590 | 387.184 | 340.227 | M-H | NA |
| V50 |  | Cer-NS d26:4 | 1.242 | 0.045 | 1.200 | 464.345 | 728.020 | M+FA-H | NA |
| V51 |  | PG 14:0 | 1.764 | 0.020 | 1.446 | 469.230 | 409.676 | M-H | Glycerophospholipid metabolism |
| V52 |  | LPG 18:3 | 1.828 | 0.033 | 1.310 | 505.266 | 349.189 | M-H | Glycerophospholipid metabolism |
| V53 |  | PS 16:0 | 1.283 | 0.011 | 1.743 | 510.253 | 411.407 | M-H | Glycerophospholipid metabolism |
| V54 |  | PC 12:0 | 1.227 | 0.034 | 1.480 | 512.258 | 411.440 | M+Hac-H | Glycerophospholipid metabolism; Linoleic acid metabolism; Arachidonic acid metabolism |
| V55 |  | PA 23:2 | 0.828 | 0.029 | 1.325 | 517.302 | 469.135 | M-H | Glycerophospholipid metabolism |
| V56 |  | PE 21:5 | 1.449 | 0.013 | 1.647 | 526.249 | 372.473 | M-H | Glycerophospholipid metabolism |
| V57 |  | PS 18:3 | 1.393 | 0.001 | 1.880 | 532.236 | 411.227 | M-H | Glycerophospholipid metabolism |
| V58 |  | Viridicatumtoxin | 1.477 | 0.008 | 1.715 | 564.194 | 252.806 | M-H | NA |
| V59 |  | Biliverdin | 1.383 | 0.025 | 1.365 | 581.242 | 933.480 | M-H | Porphyrin and chlorophyll metabolism |
| V60 |  | PI 18:4 | 1.206 | 0.035 | 1.431 | 605.240 | 358.163 | M-H | Glycerophospholipid metabolism; Glycosylphosphatidylinositol (GPI)-anchor biosynthesis; Phosphatidylinositol signaling system; Inositol phosphate metabolism |
| V61 |  | PA 30:7 | 1.205 | 0.018 | 1.507 | 605.333 | 399.543 | M-H | Glycerophospholipid metabolism; Glycerolipid metabolism |
| V62 |  | MGDG 22:3 | 1.414 | 0.010 | 1.617 | 629.356 | 342.091 | M+FA-H | Glycerolipid metabolism |
| V63 |  | DGDG 19:4 | 0.666 | 0.041 | 1.346 | 747.343 | 461.892 | M+FA-H | Glycerolipid metabolism |
| V64 |  | PE 48:8 | 1.457 | 0.034 | 1.271 | 898.638 | 728.643 | M-H | Glycerophospholipid metabolism; Glycosylphosphatidylinositol (GPI)-anchor biosynthesis |

Note: NA, Not Available; m/z, mass-to-charge ratio; Rt, retention time, FC, fold change; VIP, variable importance for the projection.

**Supplementary Table 3.** **Identification data of metabolites with significant differences between the RCC and HC groups and the metabolic pathways involved.**

| **varID** | **Scan mode** | **Compounds** | **FC** | ***P*** | **VIP** | **m/z** | **Rt(s)** | **Adducts** | **pathway** |
| --- | --- | --- | --- | --- | --- | --- | --- | --- | --- |
| V1 | ESI+ | 3,4-Dehydrothiomorpholine-3-carboxylate | 0.650 | ＜0.001 | 2.006 | 146.027 | 74.126 | M+H | NA |
| V2 |  | Benzylideneacetone | 2.134 | ＜0.001 | 2.430 | 147.080 | 383.865 | M+H | NA |
| V3 |  | Glutamic acid | 1.533 | ＜0.001 | 2.171 | 148.061 | 54.102 | M+H | D-Glutamine and D-glutamate metabolism |
| V4 |  | 4-Methoxyphenyl isothiocyanate | 1.710 | 0.002 | 1.722 | 166.035 | 382.256 | M+H | NA |
| V5 |  | Safynol | 2.358 | ＜0.001 | 2.326 | 201.091 | 464.076 | M+H | NA |
| V6 |  | 2-Oxo-9-methylthiononanoic acid | 1.937 | ＜0.001 | 2.226 | 219.102 | 463.643 | M+H | NA |
| V7 |  | Leu-Pro | 2.659 | 0.010 | 1.381 | 229.154 | 62.832 | M+H | NA |
| V8 |  | Pro-Leu | 12.390 | 0.049 | 1.089 | 229.154 | 105.848 | M+H | NA |
| V9 |  | 9,12,15-Octadecatrien-1-ol | 1.507 | 0.028 | 1.212 | 265.252 | 747.852 | M+H | NA |
| V10 |  | Geranyl-hydroxybenzoate | 2.160 | ＜0.001 | 2.353 | 275.164 | 463.820 | M+H | NA |
| V11 |  | Gingerdione | 1.963 | ＜0.001 | 2.207 | 293.175 | 464.072 | M+H | NA |
| V12 |  | (6S)-Hydroxyhyoscyamine | 1.693 | ＜0.001 | 2.508 | 306.173 | 274.650 | M+H | NA |
| V13 |  | Neryl glucoside | 2.232 | ＜0.001 | 2.522 | 317.196 | 452.039 | M+H | NA |
| V14 |  | N,N-dimethyl-Safingol | 0.446 | 0.007 | 1.435 | 330.336 | 438.008 | M+H | NA |
| V15 |  | All-cis-4,7,10,13,16-docosapentaenoic acid | 1.745 | ＜0.001 | 2.153 | 331.265 | 727.270 | M+H | NA |
| V16 |  | Oleoyl glycine | 1.530 | 0.033 | 1.111 | 340.293 | 747.577 | M+H | NA |
| V17 |  | 21-Deoxycortisol | 1.651 | ＜0.001 | 2.155 | 347.224 | 674.286 | M+H | Steroid hormone biosynthesis |
| V18 |  | 17α,21-Dihydroxypregnenolone | 2.119 | ＜0.001 | 2.722 | 349.237 | 698.951 | M+H | Steroid hormone biosynthesis |
| V19 |  | strychnine N-oxide | 1.965 | ＜0.001 | 2.261 | 351.178 | 472.795 | M+H | NA |
| V20 |  | Vincamine | 1.988 | ＜0.001 | 1.844 | 355.202 | 454.359 | M+H | NA |
| V21 |  | MG (18:1) | 1.613 | 0.024 | 1.167 | 357.300 | 747.538 | M+H | Glycerolipid metabolism |
| V22 |  | Monooleoylglycerol | 1.954 | ＜0.001 | 2.452 | 357.300 | 887.556 | M+H | NA |
| V23 |  | MG(18:1/0:0/0:0)[rac] | 1.589 | 0.014 | 1.306 | 357.299 | 725.782 | M+H | NA |
| V24 |  | 6-Keto-prostaglandin F1α | 2.801 | ＜0.001 | 3.042 | 371.243 | 668.909 | M+H | NA |
| V25 |  | [12]-Gingerol | 1.593 | 0.008 | 1.391 | 379.284 | 653.217 | M+H | NA |
| V26 |  | Dehydrocholic acid | 1.683 | ＜0.001 | 2.205 | 403.248 | 794.658 | M+H | One carbon pool by folate; Glycine, serine and threonine metabolism |
| V27 |  | Cholic Acid Methyl Ester | 1.505 | 0.006 | 1.447 | 405.301 | 876.229 | M+H-H2O | NA |
| V28 |  | Nigakilactone C | 1.432 | 0.017 | 1.324 | 435.235 | 454.609 | M+H | NA |
| V29 |  | 2-(4-Allyl-2,6-dimethoxyphenoxy)-1-(3,4,5-trimethoxyphenyl)-1-propanol | 0.526 | ＜0.001 | 2.258 | 436.238 | 454.570 | M+NH4 | NA |
| V30 |  | Limonin | 1.824 | 0.001 | 1.815 | 471.199 | 259.599 | M+H | NA |
| V31 |  | LPC 19:2 | 0.441 | ＜0.001 | 3.239 | 556.347 | 440.903 | M+Na | Glycerophospholipid metabolism |
| V32 |  | Canthaxanthin | 0.631 | 0.012 | 1.406 | 565.404 | 1080.880 | M+H | NA |
| V33 |  | Biliverdin | 0.644 | 0.005 | 1.559 | 583.255 | 427.503 | M+H | Porphyrin and chlorophyll metabolism |
| V34 |  | HexCer-AP t32:2 | 1.963 | ＜0.001 | 2.468 | 702.516 | 894.591 | M+H | NA |
| V35 |  | PC 34:2 | 0.650 | 0.014 | 1.252 | 780.552 | 1057.660 | M+Na | Glycerophospholipid metabolism; Linoleic acid metabolism; Arachidonic acid metabolism; alpha-Linolenic acid metabolism |
| V36 |  | PC(38:8) | 1.547 | 0.014 | 1.294 | 782.569 | 1056.915 | M+H | Glycerophospholipid metabolism; Linoleic acid metabolism; Arachidonic acid metabolism; alpha-Linolenic acid metabolism |
| V37 |  | Choline | 1.328 | ＜0.001 | 2.069 | 104.108 | 52.113 | M+H | Glycerophospholipid metabolism; Glycine, serine and threonine metabolism |
| V38 |  | Cinnamic acid | 1.219 | ＜0.001 | 1.961 | 131.049 | 255.047 | M+H-H2O | NA |
| V39 |  | trans-Cinnamic acid | 1.258 | ＜0.001 | 2.038 | 149.059 | 254.882 | M+H | NA |
| V40 |  | 6-Phenyl-3-hexen-2-one | 1.361 | 0.014 | 1.293 | 175.107 | 106.201 | M+H | NA |
| V41 |  | 6-(Pentylthio)purine | 1.388 | ＜0.001 | 1.928 | 223.107 | 275.009 | M+H | NA |
| V42 |  | (5R,8aR)-8-methyl-5-((E)-non-6-en-8-yn-1-yl)octahydroindolizine | 1.489 | 0.003 | 1.644 | 260.236 | 406.794 | M+H | NA |
| V43 |  | γ-Glu-Leu | 1.484 | ＜0.001 | 2.150 | 261.144 | 277.433 | M+H | NA |
| V44 |  | 9Z,12Z,15Z-Octadecatrienal | 1.437 | 0.038 | 1.115 | 263.237 | 664.074 | M+H | NA |
| V45 |  | 5-(1-hydroxypropan-2-yl)isolongifol-4-ene | 1.258 | 0.044 | 1.044 | 263.237 | 640.756 | M+H | NA |
| V46 |  | SB-206553 hydrochloride158942-04-2 | 0.800 | 0.032 | 1.149 | 293.139 | 320.321 | M+H | NA |
| V47 |  | Aspartame | 1.327 | 0.020 | 1.379 | 295.130 | 284.011 | M+H | NA |
| V48 |  | 17-α-Methyltestosterone | 1.369 | 0.015 | 1.331 | 303.232 | 650.503 | M+H | NA |
| V49 |  | Cis-8,11,14-Eicosatrienoic acid | 1.317 | 0.005 | 1.513 | 307.263 | 763.075 | M+H | Biosynthesis of unsaturated fatty acids |
| V50 |  | D-Maltose | 1.325 | 0.010 | 1.322 | 325.113 | 53.733 | M+H-H2O | Starch and sucrose metabolism |
| V51 |  | beta-D-Glucopyranoside, 4-(3-hydroxypropyl)-2-methoxyphenyl | 1.230 | 0.031 | 1.121 | 362.171 | 714.010 | M+NH4 | NA |
| V52 |  | cis-5-Tetradecenoylcarnitine | 1.284 | 0.038 | 1.178 | 370.295 | 403.656 | M+H | NA |
| V53 |  | N-Docosahexaenoyl GABA | 1.432 | 0.017 | 1.324 | 414.301 | 374.665 | M+H | Biosynthesis of unsaturated fatty acids |
| V54 |  | 6-[(1E,3E,5E)-6-[(2R,3R,3aR,4R,5R,6aS)-3,4-dihydroxy-2,3,3a-trimethyl-2,4,5,6a-tetrahydrofuro[2,3-b]furan-5-yl]hexa-1,3,5-trienyl]-4-methoxy-5-methylpyran-2-one | 1.237 | 0.010 | 1.693 | 422.222 | 939.916 | M+NH4 | NA |
| V55 |  | Lovastatin acid (Mevinolinic acid) | 1.346 | 0.034 | 1.186 | 423.270 | 757.825 | M+H | NA |
| V56 |  | Fludrocortisone acetate | 0.754 | 0.025 | 1.184 | 423.211 | 584.896 | M+H | Steroid hormone biosynthesis |
| V57 |  | (E,2S,3R,4R,5S)-2-acetamido-3,4,5,14-tetrahydroxyicos-6-enoic acid | 1.424 | ＜0.001 | 2.008 | 432.295 | 613.711 | M+H | NA |
| V58 |  | Decaline | 1.280 | 0.022 | 1.189 | 438.236 | 294.693 | M+H | NA |
| V59 |  | 13'-Hydroxy-α-tocopherol | 1.337 | 0.019 | 1.207 | 447.380 | 1108.125 | M+H | NA |
| V60 |  | LPC 18:3 | 0.751 | 0.009 | 1.445 | 518.324 | 438.162 | M+H | Glycerophospholipid metabolism |
| V61 |  | 1-Linoleoylglycerophosphocholine | 0.833 | 0.007 | 1.551 | 520.339 | 467.173 | M+H | Glycerophospholipid metabolism |
| V62 |  | Trilobolide | 0.818 | 0.029 | 1.286 | 523.246 | 636.431 | M+H | NA |
| V63 |  | [1-[3-(3-hydroxy-2,2-dimethyl-1,2'-dioxospiro[3aH-imidazo[1,2-a]indole-4,5'-oxolane]-3'-yl)-4-oxoquinazolin-2-yl]-2-methylpropyl] acetate | 1.373 | 0.001 | 1.707 | 547.219 | 429.057 | M+H | NA |
| V64 |  | Cer 35:0 | 0.759 | 0.037 | 1.152 | 554.551 | 825.432 | M+H | NA |
| V65 |  | DG 36:7 | 0.814 | 0.044 | 1.146 | 617.475 | 902.286 | M+Li | NA |
| V66 |  | DG 36:6 | 0.732 | 0.007 | 1.355 | 619.490 | 925.819 | M+Li | NA |
| V67 |  | DG 37:8 | 1.389 | 0.021 | 1.195 | 640.500 | 1101.410 | M+NH4 | NA |
| V68 |  | SM d36:2 | 1.446 | 0.002 | 1.519 | 729.591 | 1118.405 | M+H | NA |
| V69 |  | PC(16:0/18:2) | 1.375 | 0.030 | 1.294 | 758.569 | 315.971 | M+H | Glycerophospholipid metabolism; Linoleic acid metabolism; Arachidonic acid metabolism; alpha-Linolenic acid metabolism |
| V70 |  | PC(36:4) | 0.782 | 0.037 | 1.075 | 782.569 | 1056.915 | M+H | Glycerophospholipid metabolism; Linoleic acid metabolism; Arachidonic acid metabolism; alpha-Linolenic acid metabolism |
| V71 |  | plasmenyl-PC 36:1 | 1.308 | 0.028 | 1.280 | 794.606 | 1117.090 | M+Na | NA |
| V72 |  | PC(38:6) | 0.734 | 0.027 | 1.133 | 806.568 | 1058.160 | M+H | Glycerophospholipid metabolism; Linoleic acid metabolism; Arachidonic acid metabolism; alpha-Linolenic acid metabolism |
| V73 |  | PC 38:4 | 0.740 | 0.034 | 1.063 | 810.601 | 1117.020 | M+H | Glycerophospholipid metabolism; Linoleic acid metabolism; Arachidonic acid metabolism; alpha-Linolenic acid metabolism |
| V74 |  | PC(18:2/22:6) | 0.758 | 0.016 | 1.263 | 830.569 | 1057.995 | M+H | Glycerophospholipid metabolism; Linoleic acid metabolism; Arachidonic acid metabolism; alpha-Linolenic acid metabolism |
| V75 |  | PC 40:8 | 1.386 | 0.019 | 1.215 | 830.567 | 729.462 | M+H | Glycerophospholipid metabolism; Linoleic acid metabolism; Arachidonic acid metabolism; alpha-Linolenic acid metabolism |
| V76 |  | PC 40:5 | 1.245 | 0.005 | 1.410 | 836.614 | 791.546 | M+H | Glycerophospholipid metabolism; Linoleic acid metabolism; Arachidonic acid metabolism; alpha-Linolenic acid metabolism |
| V77 |  | PC 42:9 | 1.242 | 0.039 | 1.055 | 856.584 | 811.318 | M+H | Glycerophospholipid metabolism; Linoleic acid metabolism; Arachidonic acid metabolism; alpha-Linolenic acid metabolism |
| V78 | ESI- | Phosphoric acid | 1.274 | 0.034 | 1.033 | 96.962 | 69.638 | M-H | NA |
| V79 |  | 2-Hydroxy-3-methylbutyric acid | 1.288 | 0.006 | 1.308 | 117.057 | 277.438 | M-H | NA |
| V80 |  | 2-Propylpentanoic acid | 1.299 | 0.017 | 1.201 | 143.108 | 411.130 | M-H | NA |
| V81 |  | 3-Aminopentanedioic acid | 1.418 | 0.001 | 1.600 | 146.046 | 54.447 | M-H | NA |
| V82 |  | α-Fluoro-β-ureidopropionic acid | 1.319 | 0.012 | 1.242 | 149.046 | 59.564 | M-H | NA |
| V83 |  | Dl-2-Amino-3-phosphonopropionic acid | 0.829 | 0.026 | 1.111 | 150.002 | 625.068 | M-H2O-H | NA |
| V84 |  | 2-Mercaptobenzothiazole | 0.646 | 0.018 | 1.137 | 165.979 | 378.916 | M-H | NA |
| V85 |  | N-Acetyl-L-leucine | 1.305 | 0.005 | 1.292 | 172.098 | 312.051 | M-H | NA |
| V86 |  | Myoinositol | 1.293 | 0.001 | 1.592 | 179.056 | 57.310 | M-H | Glycerophospholipid metabolism; Glycerolipid metabolism; Phosphatidylinositol signaling system |
| V87 |  | Dl-P-Hydroxyphenyl lactic acid | 1.229 | 0.017 | 1.391 | 181.051 | 281.198 | M-H | NA |
| V88 |  | (4S,8R)-8,9-Dihydroxy-p-menth-1(6)-en-2-one | 1.492 | 0.015 | 1.395 | 183.103 | 377.599 | M-H | NA |
| V89 |  | Epomediol | 1.361 | 0.022 | 1.276 | 185.119 | 396.410 | M-H | NA |
| V90 |  | 1-Hydroxy-2-Naphthoic Acid | 0.671 | 0.001 | 1.910 | 187.042 | 74.587 | M-H | NA |
| V91 |  | 5-HYDROXYINDOLEACETATE | 1.251 | 0.018 | 1.153 | 190.051 | 251.955 | M-H | NA |
| V92 |  | 3-Phenylpropyl propanoate | 0.807 | 0.004 | 1.470 | 191.108 | 430.868 | M-H | NA |
| V93 |  | Indolelactate | 1.305 | 0.009 | 1.328 | 204.066 | 329.012 | M-H | NA |
| V94 |  | Glycyl-Methionine | 1.252 | 0.023 | 1.227 | 205.069 | 328.966 | M-H | NA |
| V95 |  | N-Acetyl-D-phenylalanine | 82.962 | 0.009 | 1.238 | 206.082 | 322.063 | M-H | NA |
| V96 |  | Gly-Phe | 1.500 | ＜0.001 | 1.643 | 221.093 | 272.284 | M-H | NA |
| V97 |  | 5-Acetylamino-6-formylamino-3-methyluracil | 1.360 | 0.024 | 1.094 | 225.069 | 1117.890 | M-H | Caffeine metabolism |
| V98 |  | Ethopabate | 1.241 | 0.009 | 1.256 | 236.093 | 323.270 | M-H | NA |
| V99 |  | 2,4-Dihydroxy-7,8-dimethoxy-2H-1,4-benzoxazin-3(4H)-one | 1.254 | ＜0.001 | 1.826 | 240.061 | 407.419 | M-H | NA |
| V100 |  | Uridine | 1.296 | ＜0.001 | 1.669 | 243.063 | 59.715 | M-H | Galactose metabolism; Phosphatidylinositol signaling system |
| V101 |  | Aspartyl-Asparagine | 1.466 | 0.001 | 1.554 | 246.075 | 1117.900 | M-H | NA |
| V102 |  | FA 16:2 | 1.215 | 0.020 | 1.078 | 251.202 | 628.384 | M-H | Glycerophospholipid metabolism; Glycerolipid metabolism; Phosphatidylinositol signaling system |
| V103 |  | Hypogeic acid | 1.275 | 0.008 | 1.365 | 253.217 | 698.377 | M-H | NA |
| V104 |  | Peptide 2-(3-carboxy-3-aminopropyl)-L-histidine | 1.470 | 0.001 | 1.476 | 255.114 | 342.795 | M-H | NA |
| V105 |  | Phosphotyrosine | 1.238 | 0.018 | 1.131 | 260.023 | 170.887 | M-H | NA |
| V106 |  | omega-Cyclohexylundecanoic acid | 1.232 | 0.003 | 1.436 | 267.233 | 754.696 | M-H | NA |
| V107 |  | 8-Oxohexadecanoic acid | 1.287 | 0.006 | 1.274 | 269.213 | 791.868 | M-H | NA |
| V108 |  | (-)-Maackiain | 0.727 | ＜0.001 | 2.001 | 283.070 | 635.398 | M-H | NA |
| V109 |  | Sakuranetin | 0.730 | ＜0.001 | 1.974 | 285.068 | 635.371 | M-H | NA |
| V110 |  | 16-Hydroxy-10-oxohexadecanoic acid | 1.271 | 0.001 | 1.542 | 285.207 | 615.258 | M-H | NA |
| V111 |  | (±)-Octanoylcarnitine | 1.255 | 0.002 | 1.408 | 286.211 | 615.265 | M-H | NA |
| V112 |  | 6-Hydroxypentadecanedioic acid | 1.346 | ＜0.001 | 1.726 | 287.186 | 443.238 | M-H | NA |
| V113 |  | (R)-2-Hydroxystearate | 1.209 | 0.036 | 1.065 | 299.259 | 574.652 | M-H | NA |
| V114 |  | N-Acetylgalactosamine 6-sulfate | 1.412 | ＜0.001 | 1.806 | 300.036 | 57.325 | M-H | NA |
| V115 |  | (5Z,8Z,11Z,14Z,17Z)-Icosapentaenoic acid | 1.338 | 0.031 | 1.045 | 301.217 | 649.841 | M-H | Biosynthesis of unsaturated fatty acids |
| V116 |  | (2R,3R)-3,5-dihydroxy-2-(4-hydroxyphenyl)-7-methoxy-2,3-dihydrochromen-4-one | 1.375 | 0.002 | 1.488 | 301.120 | 385.934 | M-H | NA |
| V117 |  | FTY720 | 1.266 | ＜0.001 | 1.701 | 306.252 | 762.268 | M-H | NA |
| V118 |  | N-Acetylneuraminate | 1.462 | 0.002 | 1.714 | 309.108 | 257.631 | M-H | Amino sugar and nucleotide sugar metabolism |
| V119 |  | Oleic acid methyl ester | 1.408 | ＜0.001 | 1.633 | 309.280 | 911.833 | M-H | NA |
| V120 |  | Lansamide 4 | 1.346 | ＜0.001 | 1.839 | 310.151 | 107.224 | M-H | NA |
| V121 |  | (±)13-Azaprostanoic acid | 1.371 | 0.001 | 1.574 | 310.283 | 911.721 | M-H | NA |
| V122 |  | Avocadyne 4-acetate | 1.228 | 0.012 | 1.261 | 325.239 | 728.682 | M-H | NA |
| V123 |  | (5Z,8Z,11Z,14Z,17Z)-Eicosapentaenoic acid ethyl ester | 1.297 | 0.005 | 1.369 | 329.249 | 730.598 | M-H | NA |
| V124 |  | FA 22:4 | 1.335 | ＜0.001 | 1.797 | 331.264 | 795.492 | M-H | NA |
| V125 |  | MEDICA 16 | 0.754 | 0.015 | 1.160 | 341.270 | 605.707 | M-H | NA |
| V126 |  | Melibiitol | 1.211 | 0.026 | 1.177 | 343.128 | 276.314 | M-H | Galactose metabolism |
| V127 |  | N-(Carbethoxyacetyl)-4-chloro-L-tryptophan | 1.289 | 0.001 | 1.816 | 351.078 | 107.592 | M-H | NA |
| V128 |  | Biflorin | 0.759 | 0.008 | 1.447 | 353.090 | 55.042 | M-H | NA |
| V129 |  | Tetrahydropentoxyline | 1.334 | ＜0.001 | 2.051 | 365.135 | 251.435 | M-H | NA |
| V130 |  | 5β-Androstan-3α-ol-17-one sulfate | 1.379 | 0.017 | 1.227 | 369.175 | 495.728 | M-H | NA |
| V131 |  | Biocytin | 1.325 | 0.043 | 1.026 | 371.175 | 374.660 | M-H | Glycerophospholipid metabolism; Phosphatidylinositol signaling system; Glycosylphosphatidylinositol (GPI)-anchor biosynthesis; Inositol phosphate metabolism |
| V132 |  | N6-Succinyl adenosine | 1.342 | ＜0.001 | 1.999 | 382.100 | 261.691 | M-H | NA |
| V133 |  | Dihydrozeatin-O-glucoside | 1.200 | 0.031 | 1.206 | 382.176 | 809.925 | M-H | NA |
| V134 |  | HU-211 | 1.203 | 0.029 | 1.251 | 385.271 | 421.739 | M-H | NA |
| V135 |  | D-Isoleucine | 0.667 | 0.016 | 1.197 | 392.289 | 430.560 | 3M-H | NA |
| V136 |  | dimethoxy Curcumin | 1.302 | 0.021 | 1.319 | 395.154 | 390.833 | M-H | NA |
| V137 |  | Hydrocortisone | 1.321 | 0.027 | 1.030 | 397.169 | 384.600 | M+Cl | Steroid hormone biosynthesis |
| V138 |  | JWH 210 N-(5-carboxypentyl) metabolite | 1.399 | 0.004 | 1.331 | 398.173 | 384.402 | M-H | NA |
| V139 |  | Melleolide | 0.681 | 0.007 | 1.296 | 399.183 | 584.310 | M-H | NA |
| V140 |  | Merodesmosine | 0.706 | 0.037 | 1.107 | 401.233 | 664.920 | M-H | NA |
| V141 |  | 1-(4-Hydroxy-3,5-dimethoxyphenyl)-7-(4-hydroxy-3-methoxyphenyl)-3,5-heptanediol | 0.785 | ＜0.001 | 1.677 | 405.192 | 358.404 | M-H | NA |
| V142 |  | 4'-Methylliquiritigenin 7-rhamnoside | 1.410 | 0.005 | 1.374 | 415.150 | 298.802 | M-H | NA |
| V143 |  | 1,4-Bis(2-ethylhexyl) sulfosuccinate | 0.753 | 0.013 | 1.511 | 421.227 | 899.532 | M-H | NA |
| V144 |  | Obtusifolin 2-glucoside | 1.336 | 0.001 | 1.584 | 445.120 | 252.739 | M-H | NA |
| V145 |  | Spirotaccagenin | 0.788 | 0.038 | 1.085 | 445.296 | 433.018 | M-H | NA |
| V146 |  | 2,3-Dihydro-5,5',7,7'-tetrahydroxy-2-(4-hydroxyphenyl)[3,8'-bi-4H-1-benzopyran]-4,4'-dione | 1.418 | ＜0.001 | 1.710 | 447.065 | 640.698 | M-H | NA |
| V147 |  | (ent-2b,4S,9a)-2,4,9-Trihydroxy-10(14)-oplopen-3-one 2-(2-methylbutanoate) 9-(3-methyl-2E-pentenoate) | 1.290 | 0.029 | 1.276 | 447.275 | 457.997 | M-H | NA |
| V148 |  | Hexadecanoyl-hydroxy-sn-glyceroethanolamine (PUT) | 1.214 | ＜0.001 | 1.597 | 452.278 | 487.953 | M-H | NA |
| V149 |  | LPE 16:0 | 1.200 | 0.001 | 1.540 | 452.278 | 474.676 | M-H | Glycerophospholipid metabolism |
| V150 |  | 27-Norcholestanehexol | 0.790 | 0.034 | 1.053 | 453.322 | 515.356 | M-H | NA |
| V151 |  | LPA 20:0 | 1.459 | 0.009 | 1.225 | 465.304 | 1040.590 | M-H | Glycerophospholipid metabolism; Glycerolipid metabolism; Phosphatidylinositol signaling system |
| V152 |  | Buprenorphine | 1.217 | 0.008 | 1.401 | 466.294 | 526.588 | M-H | NA |
| V153 |  | Cer-AP t27:2 | 0.833 | 0.024 | 1.071 | 468.378 | 782.176 | M-H | NA |
| V154 |  | 5-Formiminotetrahydrofolate | 1.385 | 0.003 | 1.464 | 471.171 | 263.206 | M-H | One carbon pool by folate; Glycine, serine and threonine metabolism |
| V155 |  | LPE 18:0 | 1.226 | ＜0.001 | 1.646 | 480.310 | 572.512 | M-H | Glycerophospholipid metabolism |
| V156 |  | LPE 18:0 | 1.203 | 0.001 | 1.555 | 480.310 | 554.521 | M-H | Glycerophospholipid metabolism |
| V157 |  | Sarcodon scabrosus Depsipeptide | 0.825 | 0.031 | 1.011 | 484.269 | 393.289 | M-H | NA |
| V158 |  | CerP 25:1 | 0.833 | 0.002 | 1.431 | 490.331 | 535.818 | M-H | NA |
| V159 |  | (3b,4b,11b,14b)-11-Ethoxy-3,4-epoxy-14-hydroxy-12-cyathen-15-al 14-xyloside | 0.773 | 0.017 | 1.170 | 493.283 | 433.990 | M-H | NA |
| V160 |  | Taurodeoxycholic acid | 1.354 | 0.024 | 1.073 | 498.280 | 372.339 | M-H | NA |
| V161 |  | PS 16:0 | 1.280 | 0.005 | 1.415 | 510.253 | 411.407 | M-H | Glycerophospholipid metabolism |
| V162 |  | PC 12:0 | 1.307 | 0.003 | 1.439 | 512.258 | 411.440 | M+Hac-H | Glycerophospholipid metabolism; Linoleic acid metabolism; Arachidonic acid metabolism; alpha-Linolenic acid metabolism |
| V163 |  | Cer-NP t31:0 | 1.285 | 0.015 | 1.443 | 512.478 | 791.131 | M-H | NA |
| V164 |  | PA 23:1 | 1.440 | 0.031 | 1.009 | 517.301 | 395.467 | M-H | Glycerophospholipid metabolism; Glycerolipid metabolism; Phosphatidylinositol signaling system |
| V165 |  | Tween 20 | 1.214 | 0.010 | 1.223 | 521.337 | 579.890 | M-H | NA |
| V166 |  | LPE 22:5 | 1.291 | 0.002 | 1.403 | 526.295 | 477.586 | M-H | Glycerophospholipid metabolism |
| V167 |  | PE 21:5 | 1.483 | 0.002 | 1.598 | 526.249 | 372.473 | M-H | Glycerophospholipid metabolism; Glycosylphosphatidylinositol (GPI)-anchor biosynthesis |
| V168 |  | 16,17-Dihydro-16a,17-dihydroxygibberellin A4 17-glucoside | 1.293 | 0.030 | 1.090 | 527.214 | 349.490 | M-H | NA |
| V169 |  | PS 18:3 | 1.263 | 0.012 | 1.247 | 532.236 | 411.227 | M-H | Glycerophospholipid metabolism |
| V170 |  | FAHFA 34:2 | 1.214 | 0.004 | 1.551 | 533.456 | 791.131 | M-H | NA |
| V171 |  | Cortolone-3-glucuronide | 1.386 | 0.002 | 1.411 | 541.266 | 330.419 | M-H | NA |
| V172 |  | PE 22:1 | 0.809 | 0.003 | 1.349 | 548.337 | 485.486 | M-H | Glycerophospholipid metabolism; Glycosylphosphatidylinositol (GPI)-anchor biosynthesis |
| V173 |  | FAHFA 36:3 | 1.336 | 0.007 | 1.341 | 559.474 | 728.588 | M-H | NA |
| V174 |  | Cer-AS d35:4 | 1.278 | 0.020 | 1.267 | 560.478 | 728.588 | M-H | NA |
| V175 |  | FAHFA 36:2 | 1.260 | 0.029 | 1.081 | 561.481 | 728.580 | M-H | NA |
| V176 |  | (11E)-Octadecenoic acid | 1.248 | 0.028 | 1.323 | 563.505 | 810.331 | 2M-H | NA |
| V177 |  | PI 16:0 | 0.694 | 0.002 | 1.779 | 585.265 | 357.735 | M-H | Glycerophospholipid metabolism; Phosphatidylinositol signaling system; Glycosylphosphatidylinositol (GPI)-anchor biosynthesis; Inositol phosphate metabolism |
| V178 |  | LPI 18:5 | 1.361 | 0.014 | 1.183 | 589.233 | 306.311 | M-H | Glycerophospholipid metabolism |
| V179 |  | Urobilin | 1.496 | 0.035 | 1.141 | 589.304 | 328.262 | M-H | NA |
| V180 |  | LPG 24:0 | 1.242 | 0.022 | 1.097 | 595.401 | 791.090 | M-H | Glycerophospholipid metabolism |
| V181 |  | Amphibine H | 1.430 | 0.001 | 1.848 | 604.318 | 292.357 | M-H | NA |
| V182 |  | PI 18:4 | 0.671 | ＜0.001 | 1.992 | 605.240 | 358.163 | M-H | Glycerophospholipid metabolism; Phosphatidylinositol signaling system; Glycosylphosphatidylinositol (GPI)-anchor biosynthesis; Inositol phosphate metabolism |
| V183 |  | PE 26:0 | 0.798 | 0.005 | 1.381 | 606.416 | 665.581 | M-H | Glycerophospholipid metabolism; Glycosylphosphatidylinositol (GPI)-anchor biosynthesis |
| V184 |  | Cholestane-3,7,12,25-tetrol-3-glucuronide | 1.456 | 0.024 | 1.136 | 611.381 | 388.824 | M-H | NA |
| V185 |  | LPG 26:4 | 1.474 | 0.021 | 1.262 | 615.367 | 361.253 | M-H | Glycerophospholipid metabolism |
| V186 |  | Phosphatidylinositol lyso 20:4 | 1.269 | ＜0.001 | 1.656 | 619.291 | 1125.080 | M-H | NA |
| V187 |  | PC 22:2 | 0.813 | 0.006 | 1.329 | 634.375 | 541.381 | M+FA-H | Glycerophospholipid metabolism; Linoleic acid metabolism; Arachidonic acid metabolism; alpha-Linolenic acid metabolism |
| V188 |  | Coproporphyrin I | 1.462 | 0.034 | 1.002 | 653.268 | 301.632 | M-H | NA |
| V189 |  | Neoacrimarine A | 1.224 | 0.025 | 1.076 | 680.284 | 309.399 | M-H | NA |
| V190 |  | SM d34:1 | 1.362 | 0.015 | 1.152 | 747.569 | 1128.410 | M+FA-H | NA |
| V191 |  | MGDG 38:5 | 1.249 | 0.024 | 1.049 | 803.567 | 504.377 | M-H | Glycerolipid metabolism |
| V192 |  | PC 36:2 | 1.470 | 0.001 | 1.565 | 830.595 | 452.816 | M+FA-H | Glycerophospholipid metabolism; Linoleic acid metabolism; Arachidonic acid metabolism; alpha-Linolenic acid metabolism |
| V193 |  | 10-DEACETYL-7-XYLOTAXOL | 1.385 | 0.005 | 1.304 | 942.349 | 263.206 | M-H | NA |
| V194 |  | HexCer-NS d53:4 | 1.446 | 0.002 | 1.523 | 958.810 | 791.196 | M-H | NA |
| V195 |  | Disialosyl galactosyl globoside | 1.348 | 0.017 | 1.181 | 964.332 | 263.361 | M-H | NA |
| V196 |  | HexCer-NS d55:5 | 1.348 | 0.017 | 1.181 | 984.827 | 810.468 | M-H | NA |
| V197 |  | Hydroiodic acid | 2.593 | 0.024 | 1.071 | 126.905 | 253.193 | M-H | One carbon pool by folate; Glycine, serine and threonine metabolism |
| V198 |  | 2,4-Dihydroxybenzoic Acid | 0.646 | 0.018 | 1.137 | 153.020 | 315.740 | M-H | NA |
| V199 |  | Acetyleugenol | 82.962 | 0.009 | 1.238 | 205.088 | 389.923 | M-H | NA |
| V200 |  | 3,4-Dihydro-6-methoxy-2,2-dimethyl-2H-1-benzopyran-4-ol | 11.150 | 0.021 | 1.137 | 207.103 | 399.779 | M-H | NA |
| V201 |  | γ-Aminobutyryl-lysine | 0.485 | ＜0.001 | 3.687 | 230.155 | 545.151 | M-H | NA |
| V202 |  | Leucyl-leucine | 2.024 | ＜0.001 | 1.892 | 243.170 | 291.122 | M-H | NA |
| V203 |  | Isoleucyl-Hydroxyproline | 1.721 | 0.001 | 1.559 | 243.135 | 307.117 | M-H | NA |
| V204 |  | L-β-aspartyl-L-leucine | 1.638 | 0.001 | 1.820 | 245.114 | 248.173 | M-H | NA |
| V205 |  | 3-Hydroxydodecanedioic acid | 1.741 | 0.014 | 1.502 | 245.139 | 380.510 | M-H | NA |
| V206 |  | 3-β-D-Galactosyl-sn-glycerol | 0.322 | 0.000 | 3.853 | 253.083 | 481.035 | M-H | Galactose metabolism |
| V207 |  | 7,8-Dihydroneopterin | 0.501 | 0.000 | 3.167 | 254.086 | 481.173 | M-H | Folate biosynthesis |
| V208 |  | Pentachlorophenol | 1.238 | 0.018 | 1.131 | 262.840 | 509.154 | M-H | NA |
| V209 |  | Ethylene brassylate | 1.885 | ＜0.001 | 2.163 | 269.176 | 456.270 | M-H | NA |
| V210 |  | 3-Hydroxytetradecanedioic acid | 1.604 | 0.002 | 1.492 | 273.169 | 395.586 | M-H | NA |
| V211 |  | 4-Hydroxy-5-(3'-hydroxyphenyl)-valeric acid-3'-O-sulphate | 3.822 | 0.015 | 1.479 | 289.045 | 329.387 | M-H | NA |
| V212 |  | γ-Glutamylphenylalanine | 1.932 | 0.006 | 1.659 | 293.114 | 283.958 | M-H | NA |
| V213 |  | Leu-Ala-OH | 0.583 | ＜0.001 | 2.060 | 309.101 | 380.336 | M-H | Pyrimidine metabolism; Pantothenate and CoA biosynthesis; beta-Alanine metabolism |
| V214 |  | Phytanate | 0.417 | 0.045 | 1.164 | 311.295 | 936.888 | M-H | NA |
| V215 |  | Glutamyltryptophan | 1.686 | 0.015 | 1.266 | 332.125 | 271.527 | M-H | NA |
| V216 |  | Erucic acid | 1.865 | 0.006 | 1.478 | 337.311 | 1026.010 | M-H | NA |
| V217 |  | Desmethylxanthohumol | 2.271 | 0.001 | 1.543 | 340.140 | 261.259 | M-H | NA |
| V218 |  | Propofol glucuronide | 92.269 | 0.007 | 1.368 | 353.160 | 370.217 | M-H | NA |
| V219 |  | Prostaglandin E1 | 3.417 | 0.014 | 1.216 | 353.233 | 404.588 | M-H | NA |
| V220 |  | 2-acetoxy-6-pentadecylbenzoic acid | 0.593 | 0.003 | 1.384 | 389.270 | 476.100 | M-H | NA |
| V221 |  | Latanoprost (free acid)-d4 | 1.901 | 0.007 | 1.282 | 393.265 | 381.170 | M-H | NA |
| V222 |  | Sorbitan palmitate (INN) | 1.302 | 0.021 | 1.319 | 401.291 | 746.319 | M-H | NA |
| V223 |  | L-902,688 | 1.650 | 0.002 | 1.499 | 418.215 | 373.339 | M-H | NA |
| V224 |  | PC 7:0 | 1.629 | 0.011 | 1.231 | 442.191 | 372.218 | M+Hac-H | Glycerophospholipid metabolism; Linoleic acid metabolism; Arachidonic acid metabolism; alpha-Linolenic acid metabolism |
| V225 |  | Leukotriene E4-d5 | 2.002 | ＜0.001 | 1.991 | 443.265 | 545.272 | M-H | Arachidonic acid metabolism |
| V226 |  | Estrone glucuronide | 1.581 | 0.001 | 1.782 | 445.191 | 338.307 | M-H | Steroid hormone biosynthesis |
| V227 |  | 7-Hydroxy-8-O-methylaloin B | 0.617 | ＜0.001 | 2.099 | 447.135 | 485.972 | M-H | NA |
| V228 |  | Glycoursodeoxycholic acid | 1.737 | 0.049 | 1.127 | 448.306 | 375.611 | M-H | NA |
| V229 |  | Bis-γ-glutamylcystine | 2.664 | ＜0.001 | 1.756 | 497.093 | 452.892 | M-H | NA |
| V230 |  | Leukotriene D4 methyl ester | 1.710 | 0.002 | 1.441 | 509.275 | 376.967 | M-H | NA |
| V231 |  | PA 23:2 | 1.507 | 0.026 | 1.051 | 517.301 | 395.467 | M-H | Glycerophospholipid metabolism; Glycerolipid metabolism; Phosphatidylinositol signaling system |
| V232 |  | bis(7)-Tacrine | 1.950 | 0.028 | 1.059 | 527.289 | 333.821 | M+Cl | NA |
| V233 |  | Glycochenodeoxycholate 7-sulfate | 1.620 | 0.032 | 1.265 | 528.264 | 399.809 | M-H | NA |
| V234 |  | N-[(3a,5b,7b)-7-hydroxy-24-oxo-3-(sulfooxy)cholan-24-yl]-Glycine | 3.209 | 0.048 | 1.220 | 528.264 | 361.428 | M-H | NA |
| V235 |  | (5xi,6alpha,7alpha,9xi,16xi)-16-(beta-D-Glucopyranosyloxy)-6,7,17-trihydroxykauran-19-oic acid | 1.557 | 0.029 | 1.237 | 529.268 | 399.848 | M-H | NA |
| V236 |  | Leukotriene F4 | 1.570 | 0.017 | 1.376 | 567.280 | 267.433 | M-H | NA |
| V237 |  | 10-Acetoxyligustroside | 1.536 | ＜0.001 | 2.004 | 581.185 | 252.756 | M-H | NA |
| V238 |  | MGDG 22:3 | 1.547 | ＜0.001 | 1.757 | 629.356 | 342.091 | M+FA-H | Glycerolipid metabolism |
| V239 |  | PS 41:7 | 1.525 | 0.001 | 1.616 | 846.520 | 471.236 | M-H | Glycerophospholipid metabolism |
| V240 |  | PE 48:8 | 1.536 | 0.017 | 1.333 | 898.638 | 728.643 | M-H | Glycerophospholipid metabolism; Glycosylphosphatidylinositol (GPI)-anchor biosynthesis |

Note: NA, Not Available; m/z, mass-to-charge ratio; Rt, retention time, FC, fold change; VIP, variable importance for the projection.

**Supplementary Table 4. Identification data of metabolites with significant differences between the BKT and HC groups and the metabolic pathways involved.**

| **varID** | **Scan mode** | **Compounds** | **FC** | ***P*** | **VIP** | **m/z** | **Rt(s)** | **Adducts** | **pathway** |
| --- | --- | --- | --- | --- | --- | --- | --- | --- | --- |
| V1 | ESI+ | Stachydrine | 2.111 | 0.006 | 1.576 | 144.102 | 59.386 | M+H | NA |
| V2 |  | 3,4-Dehydrothiomorpholine-3-carboxylate | 0.632 | ＜0.001 | 1.987 | 146.027 | 74.126 | M+H | NA |
| V3 |  | Benzylideneacetone | 1.793 | ＜0.001 | 2.406 | 147.080 | 383.865 | M+H | NA |
| V4 |  | 4-Methoxyphenyl isothiocyanate | 1.592 | 0.007 | 1.687 | 166.035 | 382.256 | M+H | NA |
| V5 |  | 3,4-Dihydroxyphenylacetate | 0.469 | 0.003 | 1.537 | 169.049 | 367.975 | M+H | Tyrosine metabolism |
| V6 |  | 2-Ethoxynaphthalene | 1.590 | 0.001 | 2.265 | 173.096 | 698.523 | M+H | NA |
| V7 |  | 6-Phenyl-3-hexen-2-one | 1.591 | 0.001 | 1.943 | 175.107 | 106.201 | M+H | NA |
| V8 |  | 2,5,6-Trihydroxy-5,6-dihydroquinoline | 0.517 | 0.048 | 1.054 | 180.065 | 302.213 | M+H | NA |
| V9 |  | Safynol | 1.892 | 0.001 | 2.092 | 201.091 | 464.076 | M+H | NA |
| V10 |  | (+)-(3S,4R)-cis-3,4-Dihydroxy-3,4-dihydrofluorene | 2.075 | ＜0.001 | 2.722 | 201.091 | 698.910 | M+H | NA |
| V11 |  | 2-Oxo-9-methylthiononanoic acid | 1.503 | 0.010 | 1.691 | 219.102 | 463.643 | M+H | NA |
| V12 |  | Ethiofencarb | 1.856 | 0.001 | 2.237 | 243.122 | 451.777 | M+NH4 | NA |
| V13 |  | (E)-4-((3aS)-1,8-dimethyl-2,3,8,8a-tetrahydropyrrolo[2,3-b]indol-3a(1H)-yl)-2-methylbut-2-en-1-ol | 1.618 | 0.001 | 2.155 | 273.206 | 611.919 | M+H | NA |
| V14 |  | 1b-Furanoeudesm-4(15)-en-1-ol acetate | 2.071 | ＜0.001 | 2.641 | 275.164 | 698.853 | M+H | NA |
| V15 |  | Geranyl-hydroxybenzoate | 1.779 | ＜0.001 | 2.162 | 275.164 | 463.820 | M+H | NA |
| V16 |  | Oleamide | 13.296 | 0.045 | 1.604 | 282.278 | 725.954 | M+H | NA |
| V17 |  | Gingerdione | 1.588 | 0.005 | 1.798 | 293.175 | 464.072 | M+H | NA |
| V18 |  | Pentanoic acid, 3-methyl-, 5-hydroxy-3-(hydroxymethyl)-6-(1-methylethyl)-2-oxo-3-cyclohexen-1-yl ester | 0.747 | 0.029 | 1.431 | 298.310 | 856.438 | M+H | NA |
| V19 |  | (6S)-Hydroxyhyoscyamine | 1.762 | ＜0.001 | 3.105 | 306.173 | 274.650 | M+H | NA |
| V20 |  | (2S)-2-(2-hydroxypropan-2-yl)-6-(2-methylbut-3-en-2-yl)-2,3-dihydrofuro[3,2-g]chromen-7-one | 1.566 | 0.003 | 1.950 | 315.157 | 463.726 | M+H | NA |
| V21 |  | Neryl glucoside | 1.882 | 0.001 | 2.160 | 317.196 | 452.039 | M+H | NA |
| V22 |  | N-2-[4-(3,3-Dimethylallyloxy)phenyl]ethylcinnamide | 0.646 | 0.025 | 1.229 | 336.191 | 268.740 | M+H | NA |
| V23 |  | 21-Deoxycortisol | 1.566 | ＜0.001 | 2.242 | 347.224 | 674.286 | M+H | Steroid hormone biosynthesis |
| V24 |  | 17α,21-Dihydroxypregnenolone | 2.095 | ＜0.001 | 2.678 | 349.237 | 698.951 | M+H | Steroid hormone biosynthesis |
| V25 |  | strychnine N-oxide | 1.567 | 0.005 | 1.986 | 351.178 | 472.795 | M+H | NA |
| V26 |  | Vincamine | 1.788 | 0.007 | 1.617 | 355.202 | 454.359 | M+H | NA |
| V27 |  | Monooleoylglycerol | 1.871 | ＜0.001 | 2.311 | 357.300 | 887.556 | M+H | NA |
| V28 |  | Cetyltrimethylammonium bromide | 1.812 | ＜0.001 | 2.215 | 363.257 | 451.691 | M+H | NA |
| V29 |  | 6-Keto-prostaglandin F1α | 2.992 | ＜0.001 | 2.904 | 371.243 | 668.909 | M+H | NA |
| V30 |  | 15(R),19(R)-hydroxy Prostaglandin F1α; | 2.098 | ＜0.001 | 2.496 | 373.258 | 667.639 | M+H | NA |
| V31 |  | 2-Pentenoic acid, 3-[(acetyloxy)methyl]-5-[(1R,4aR,8aR)-decahydro-5,5,8a-trimethyl-2-methylene-1-naphthalenyl]-, methyl ester, (2Z)- | 2.093 | ＜0.001 | 2.764 | 394.296 | 698.960 | M+NH4 | NA |
| V32 |  | Dehydrocholic acid | 1.736 | ＜0.001 | 2.357 | 403.248 | 794.658 | M+H | One carbon pool by folate; Glycine, serine and threonine metabolism |
| V33 |  | 2-[5-[2-[2-[5-(2-hydroxybutyl)oxolan-2-yl]propanoyloxy]butyl]oxolan-2-yl]propanoic acid | 1.516 | 0.001 | 2.067 | 415.269 | 613.942 | M+H | NA |
| V34 |  | Lovastatin acid (Mevinolinic acid) | 1.571 | ＜0.001 | 2.086 | 423.270 | 757.825 | M+H | NA |
| V35 |  | 3β-Hydroxy-16-phosphonopregn-5-en-20-one monoethyl ester | 1.631 | 0.004 | 2.111 | 425.254 | 792.258 | M+H | NA |
| V36 |  | Nigakilactone C | 0.587 | 0.001 | 1.799 | 435.235 | 454.609 | M+H | NA |
| V37 |  | 2-(4-Allyl-2,6-dimethoxyphenoxy)-1-(3,4,5-trimethoxyphenyl)-1-propanol | 0.571 | ＜0.001 | 1.913 | 436.238 | 454.570 | M+NH4 | NA |
| V38 |  | Canthaxanthin | 0.591 | 0.005 | 1.901 | 565.404 | 1080.880 | M+H | NA |
| V39 |  | Biliverdin | 0.557 | 0.001 | 1.796 | 583.255 | 427.503 | M+H | Porphyrin and chlorophyll metabolism |
| V40 |  | HexCer-AP t32:2 | 1.562 | 0.001 | 2.167 | 702.516 | 894.591 | M+H | NA |
| V41 |  | PC 34:2 | 0.825 | 0.049 | 1.062 | 780.552 | 1057.660 | M+Na | Glycerophospholipid metabolism; Arachidonic acid metabolism; Linoleic acid metabolism; alpha-Linolenic acid metabolism |
| V42 |  | 4-oxo 2-Nonenal-d3 | 0.768 | 0.008 | 1.434 | 158.118 | 281.869 | M+H | NA |
| V43 |  | 6-(Pentylthio)purine | 1.356 | 0.001 | 1.973 | 223.107 | 275.009 | M+H | NA |
| V44 |  | Ambroxane | 1.349 | 0.007 | 1.490 | 237.221 | 699.496 | M+H | NA |
| V45 |  | (5R,8aR)-8-methyl-5-((E)-non-6-en-8-yn-1-yl)octahydroindolizine | 1.295 | 0.035 | 1.367 | 260.236 | 406.794 | M+H | NA |
| V46 |  | 5-(1-hydroxypropan-2-yl)isolongifol-4-ene | 1.367 | 0.045 | 1.956 | 263.237 | 640.756 | M+H | NA |
| V47 |  | Hexadecanoic acid | 1.363 | 0.018 | 1.672 | 274.274 | 377.559 | M+NH4 | Biosynthesis of unsaturated fatty acids; Fatty acid biosynthesis; Fatty acid elongation; Fatty acid degradation |
| V48 |  | 2-Octenoylcarnitine | 0.785 | 0.018 | 1.371 | 286.201 | 328.469 | M+H | NA |
| V49 |  | SB-206553 hydrochloride158942-04-2 | 0.702 | 0.001 | 1.649 | 293.139 | 320.321 | M+H | NA |
| V50 |  | Palmitoyl N-Isopropylamide | 0.747 | 0.029 | 1.431 | 298.310 | 856.438 | M+H | NA |
| V51 |  | 5α-Androstane-3,17-dione | 0.818 | 0.045 | 1.222 | 311.205 | 341.230 | M+Na | Steroid hormone biosynthesis |
| V52 |  | Cajanol | 0.762 | 0.017 | 1.307 | 317.096 | 458.531 | M+H | NA |
| V53 |  | D-Maltose | 1.212 | 0.023 | 1.355 | 325.113 | 53.733 | M+H-H2O | Starch and sucrose metabolism |
| V54 |  | Dehydroneotenone | 0.792 | 0.006 | 1.548 | 337.064 | 74.009 | M+H | NA |
| V55 |  | Santalyl phenylacetate | 0.833 | 0.009 | 1.520 | 339.232 | 641.427 | M+H | NA |
| V56 |  | beta-D-Glucopyranoside, 4-(3-hydroxypropyl)-2-methoxyphenyl | 1.256 | 0.019 | 1.416 | 362.171 | 714.010 | M+NH4 | NA |
| V57 |  | Cholic Acid Methyl Ester | 1.382 | 0.043 | 1.357 | 405.301 | 876.229 | M+H-H2O | NA |
| V58 |  | 4α-hydroxymethyl-5α-cholesta-8,24-dien-3β-ol | 0.739 | 0.013 | 1.354 | 415.357 | 941.116 | M+H | NA |
| V59 |  | α-Tocotrienol | 1.267 | 0.001 | 2.129 | 425.337 | 1017.010 | M+H | NA |
| V60 |  | (6R)-6,19-ethano-25-hydroxy-6,19-dihydrovitamin D3 / (6R)-6,19-ethano-25-hydroxy-6,19-dihydrocholecalciferol | 0.820 | 0.018 | 1.436 | 429.373 | 1000.018 | M+H | NA |
| V61 |  | Hecogenin | 0.828 | 0.015 | 1.372 | 431.316 | 515.665 | M+H | NA |
| V62 |  | (E,2S,3R,4R,5S)-2-acetamido-3,4,5,14-tetrahydroxyicos-6-enoic acid | 1.414 | 0.006 | 2.034 | 432.295 | 613.711 | M+H | NA |
| V63 |  | 23-Acetoxysoladulcidine | 0.758 | 0.005 | 1.484 | 474.367 | 572.560 | M+H | NA |
| V64 |  | Clobetasone 17-Butyrate | 0.821 | 0.037 | 1.310 | 479.205 | 933.683 | M+H | NA |
| V65 |  | 1-Palmitoyl-Sn-Glycero-3-Phosphocholine | 1.326 | 0.038 | 1.255 | 496.330 | 449.002 | M+H | Glycerophospholipid metabolism |
| V66 |  | lysoPC 18:3 | 0.717 | ＜0.001 | 1.861 | 518.324 | 438.162 | M+H | Glycerophospholipid metabolism |
| V67 |  | 1-Linoleoylglycerophosphocholine | 0.803 | 0.001 | 1.776 | 520.339 | 467.173 | M+H | Glycerophospholipid metabolism |
| V68 |  | LPC(18:1) | 0.814 | 0.002 | 1.684 | 522.346 | 467.896 | M+H | Glycerophospholipid metabolism |
| V69 |  | Maltotriose | 1.388 | 0.041 | 1.280 | 527.157 | 58.518 | M+Na | NA |
| V70 |  | LPC 20:4 | 1.254 | 0.028 | 1.225 | 544.349 | 581.195 | M+H | Glycerophospholipid metabolism |
| V71 |  | Cer 35:0 | 0.732 | 0.027 | 1.300 | 554.551 | 825.432 | M+H | NA |
| V72 |  | LPC 19:2 | 0.748 | 0.002 | 1.743 | 556.347 | 440.903 | M+Na | Glycerophospholipid metabolism |
| V73 |  | Campesteryl glucoside | 1.324 | 0.033 | 1.378 | 563.428 | 788.636 | M+H | NA |
| V74 |  | SM d28:2 | 1.243 | 0.002 | 1.665 | 617.468 | 469.542 | M+H | NA |
| V75 |  | DG 38:6 | 1.454 | 0.013 | 1.355 | 647.522 | 1057.480 | M+Li | NA |
| V76 |  | SM d34:2 | 0.695 | 0.001 | 1.791 | 701.559 | 754.407 | M+H | Sphingolipid metabolism |
| V77 |  | SM d36:2 | 1.316 | 0.049 | 1.091 | 729.591 | 1118.405 | M+H | Sphingolipid metabolism |
| V78 |  | PC(32:1) | 0.704 | 0.008 | 1.596 | 732.556 | 1117.370 | M+H | Glycerophospholipid metabolism; Arachidonic acid metabolism; Linoleic acid metabolism; alpha-Linolenic acid metabolism |
| V79 |  | PC(36:4) | 0.773 | 0.024 | 1.179 | 782.569 | 1056.915 | M+H | Glycerophospholipid metabolism; Arachidonic acid metabolism; Linoleic acid metabolism; alpha-Linolenic acid metabolism |
| V80 |  | plasmenyl-PC 36:1 | 1.366 | 0.008 | 1.474 | 794.606 | 1117.090 | M+Na | NA |
| V81 |  | PC(38:6) | 0.753 | 0.036 | 1.187 | 806.568 | 1058.160 | M+H | Glycerophospholipid metabolism; Arachidonic acid metabolism; Linoleic acid metabolism; alpha-Linolenic acid metabolism |
| V82 |  | PC 38:4 | 0.682 | 0.007 | 1.464 | 810.601 | 1117.020 | M+H | Glycerophospholipid metabolism; Arachidonic acid metabolism; Linoleic acid metabolism; alpha-Linolenic acid metabolism |
| V83 |  | SM d42:2 | 1.378 | 0.027 | 1.730 | 813.681 | 1117.595 | M+H | Sphingolipid metabolism |
| V84 |  | PC(18:2/22:6) | 0.667 | 0.001 | 1.729 | 830.569 | 1057.995 | M+H | Glycerophospholipid metabolism; Arachidonic acid metabolism; Linoleic acid metabolism; alpha-Linolenic acid metabolism |
| V85 |  | PC 40:8 | 1.303 | 0.049 | 1.175 | 830.567 | 729.462 | M+H | Glycerophospholipid metabolism; Arachidonic acid metabolism; Linoleic acid metabolism; alpha-Linolenic acid metabolism |
| V86 |  | Ethyl maltol | 0.573 | 0.007 | 1.528 | 139.041 | 323.292 | M-H | NA |
| V87 |  | γ-Nonalactone | 2.063 | 0.000 | 2.491 | 155.108 | 392.681 | M-H | NA |
| V88 |  | 2-Phenylbutyric acid | 2.366 | 0.000 | 2.248 | 163.077 | 384.829 | M-H | NA |
| V89 |  | Methyl 2,4,6-trihydroxybenzoate | 0.518 | 0.007 | 1.530 | 183.030 | 323.464 | M-H | NA |
| V90 |  | 1-Hydroxy-2-Naphthoic Acid | 0.623 | ＜0.001 | 2.181 | 187.042 | 74.587 | M-H | NA |
| V91 |  | 5-Pentyltetrahydro-2-oxo-3-furancarboxylic acid | 2.167 | ＜0.001 | 2.410 | 199.098 | 392.502 | M-H | NA |
| V92 |  | Leucyl-leucine | 1.954 | ＜0.001 | 2.200 | 243.170 | 291.122 | M-H | NA |
| V93 |  | Pentachlorophenol | 0.424 | 0.014 | 1.575 | 262.840 | 509.154 | M-H | NA |
| V94 |  | Phenylalanyl-Cysteine | 2.399 | ＜0.001 | 2.523 | 267.085 | 392.414 | M-H | NA |
| V95 |  | Ethylene brassylate | 1.637 | 0.003 | 1.942 | 269.176 | 456.270 | M-H | NA |
| V96 |  | 3-Hydroxytetradecanedioic acid | 1.562 | 0.004 | 1.848 | 273.169 | 395.586 | M-H | NA |
| V97 |  | α-CEHC | 0.622 | ＜0.001 | 2.266 | 277.180 | 513.793 | M-H | NA |
| V98 |  | Leu-Ala-OH | 0.633 | 0.001 | 2.019 | 309.101 | 380.336 | M-H | Pantothenate and CoA biosynthesis; beta-Alanine metabolism; Pyrimidine metabolism |
| V99 |  | 12(13)Ep-9-KODE | 1.613 | 0.022 | 1.525 | 309.204 | 410.630 | M-H | NA |
| V100 |  | Epijasminoside A | 1.973 | ＜0.001 | 2.447 | 329.161 | 450.055 | M-H | NA |
| V101 |  | Prostaglandin D2 | 0.601 | 0.004 | 1.672 | 333.206 | 426.494 | M-H2O-H | Arachidonic acid metabolism |
| V102 |  | Erucic acid | 1.689 | 0.032 | 1.316 | 337.311 | 1026.010 | M-H | NA |
| V103 |  | Desmethylxanthohumol | 1.742 | 0.018 | 1.432 | 340.140 | 261.259 | M-H | NA |
| V104 |  | (-)-Usnic acid | 0.650 | 0.006 | 1.533 | 343.086 | 352.620 | M-H | NA |
| V105 |  | 2-acetoxy-6-pentadecylbenzoic acid | 0.617 | 0.006 | 1.563 | 389.270 | 476.100 | M-H | NA |
| V106 |  | 7β,12β-Dihydroxy-5β-cholan-24-oic acid | 0.632 | 0.010 | 1.447 | 391.285 | 430.561 | M-H | NA |
| V107 |  | L-Isoleucine | 0.626 | 0.027 | 1.268 | 392.289 | 472.610 | 3M-H | Valine, leucine and isoleucine biosynthesis; Valine, leucine and isoleucine degradation; Aminoacyl-tRNA biosynthesis |
| V108 |  | D-Isoleucine | 0.645 | 0.011 | 1.449 | 392.289 | 430.560 | 3M-H | NA |
| V109 |  | Tiocarlide (INN) | 0.381 | 0.022 | 1.305 | 399.221 | 434.056 | M-H | NA |
| V110 |  | Melleolide | 0.666 | 0.007 | 1.573 | 399.183 | 584.310 | M-H | NA |
| V111 |  | 3α,7α-Dihydroxy-12-oxo-5β-cholanate | 2.295 | 0.040 | 1.353 | 405.265 | 384.719 | M-H | NA |
| V112 |  | L-902,688 | 2.388 | 0.019 | 1.617 | 418.215 | 373.339 | M-H | NA |
| V113 |  | Leukotriene E4-d5 | 1.635 | 0.002 | 1.915 | 443.265 | 545.272 | M-H | Arachidonic acid metabolism |
| V114 |  | PG 14:0 | 0.502 | 0.004 | 1.591 | 469.230 | 409.676 | M-H | Glycerophospholipid metabolism |
| V115 |  | (3b,16b,20R)-Pregn-5-ene-3,16,20-triol 3-glucoside | 0.505 | 0.045 | 1.214 | 493.283 | 433.990 | M-H | NA |
| V116 |  | Bis-γ-glutamylcystine | 1.951 | 0.016 | 1.512 | 497.093 | 452.892 | M-H | NA |
| V117 |  | Tauroursodeoxycholic acid | 1.937 | 0.037 | 1.469 | 498.290 | 421.386 | M-H | NA |
| V118 |  | Capsianoside V | 0.612 | 0.028 | 1.323 | 513.269 | 370.530 | M-H | NA |
| V119 |  | PA 23:3 | 0.335 | 0.003 | 1.671 | 515.287 | 331.818 | M-H | Glycerophospholipid metabolism; Glycerolipid metabolism; Phosphatidylinositol signaling system |
| V120 |  | Leukotriene F4 | 2.071 | 0.001 | 2.230 | 567.280 | 267.433 | M-H | NA |
| V121 |  | PI 16:0 | 0.586 | ＜0.001 | 2.249 | 585.265 | 357.735 | M-H | Glycerophospholipid metabolism; Glycosylphosphatidylinositol (GPI)-anchor biosynthesis; Phosphatidylinositol signaling system; Inositol phosphate metabolism |
| V122 |  | D-Urobilin | 1.757 | 0.049 | 1.257 | 587.289 | 328.972 | M-H | NA |
| V123 |  | PS 23:4 | 1.683 | 0.002 | 1.929 | 600.302 | 269.810 | M-H | Glycerophospholipid metabolism |
| V124 |  | PG 24:4 | 1.602 | 0.001 | 1.993 | 601.305 | 269.940 | M-H | Glycerophospholipid metabolism |
| V125 |  | PI 18:4 | 0.556 | ＜0.001 | 2.571 | 605.240 | 358.163 | M-H | Glycerophospholipid metabolism; Glycosylphosphatidylinositol (GPI)-anchor biosynthesis; Phosphatidylinositol signaling system; Inositol phosphate metabolism |
| V126 |  | Coproporphyrin I | 1.600 | 0.030 | 1.482 | 653.268 | 301.632 | M-H | NA |
| V127 | ESI- | Phosphoric acid | 1.262 | 0.035 | 1.331 | 96.962 | 69.638 | M-H | NA |
| V128 |  | Ethyl propyl disulfide | 1.283 | 0.036 | 1.285 | 135.029 | 56.059 | M-H | NA |
| V129 |  | 3-Aminopentanedioic acid | 1.242 | 0.040 | 1.428 | 146.046 | 54.447 | M-H | NA |
| V130 |  | Cyromazine | 0.762 | 0.028 | 1.319 | 165.092 | 410.689 | M-H | NA |
| V131 |  | L-2,3-Dihydrodipicolinate | 0.814 | 0.004 | 1.700 | 168.024 | 102.753 | M-H | NA |
| V132 |  | Dodecanoic acid | 1.345 | 0.038 | 1.354 | 200.174 | 565.950 | M-H | Fatty acid biosynthesis |
| V133 |  | Gly-Phe | 1.460 | 0.001 | 1.946 | 221.093 | 272.284 | M-H | NA |
| V134 |  | Curcumenone | 1.224 | ＜0.001 | 2.545 | 233.155 | 523.757 | M-H | NA |
| V135 |  | Hypogeic acid | 1.308 | 0.015 | 1.907 | 253.217 | 698.377 | M-H | NA |
| V136 |  | Ifosfamide | 0.796 | 0.029 | 1.299 | 259.014 | 299.595 | M-H | NA |
| V137 |  | (±)-Abscisic acid | 0.776 | 0.037 | 1.305 | 263.129 | 378.725 | M-H | NA |
| V138 |  | Estrone | 0.764 | 0.023 | 1.335 | 269.150 | 331.076 | M-H | Steroid hormone biosynthesis |
| V139 |  | (-)-Maackiain | 0.781 | 0.003 | 1.840 | 283.070 | 635.398 | M-H | NA |
| V140 |  | Sakuranetin | 0.796 | 0.007 | 1.741 | 285.068 | 635.371 | M-H | NA |
| V141 |  | 6-Hydroxypentadecanedioic acid | 1.202 | 0.034 | 1.417 | 287.186 | 443.238 | M-H | NA |
| V142 |  | D-Norvaline | 1.479 | 0.034 | 1.310 | 293.175 | 420.113 | 2M+Hac-H | NA |
| V143 |  | Cis-8,11,14-Eicosatrienoic acid | 1.230 | 0.013 | 1.661 | 305.248 | 762.364 | M-H | Biosynthesis of unsaturated fatty acids |
| V144 |  | 8(R)-HPODE | 0.794 | 0.014 | 1.499 | 311.222 | 422.049 | M-H | NA |
| V145 |  | Laurolitsine | 0.693 | 0.007 | 1.592 | 312.127 | 391.976 | M-H | NA |
| V146 |  | Thiamin monophosphate | 0.801 | 0.015 | 1.479 | 343.071 | 299.894 | M-H | Thiamine metabolism |
| V147 |  | Arachidonyltrifluoromethane | 0.693 | ＜0.001 | 2.374 | 355.228 | 526.581 | M-H | NA |
| V148 |  | SB 415286 | 0.757 | 0.004 | 1.749 | 358.033 | 73.478 | M-H | NA |
| V149 |  | Rosmarinate | 0.688 | 0.004 | 1.626 | 359.081 | 392.230 | M-H | NA |
| V150 |  | HU-211 | 1.229 | 0.007 | 1.710 | 385.271 | 421.739 | M-H | NA |
| V151 |  | 7α-hydroxy-3-oxochol-4-en-24-oic Acid | 0.757 | 0.037 | 1.249 | 387.255 | 424.071 | M-H | NA |
| V152 |  | JWH 210 N-(5-carboxypentyl) metabolite | 1.323 | 0.030 | 1.324 | 398.173 | 384.402 | M-H | NA |
| V153 |  | Sorbitan palmitate (INN) | 1.405 | 0.007 | 1.663 | 401.291 | 746.319 | M-H | NA |
| V154 |  | 1-(4-Hydroxy-3,5-dimethoxyphenyl)-7-(4-hydroxy-3-methoxyphenyl)-3,5-heptanediol | 0.831 | 0.004 | 1.699 | 405.192 | 358.404 | M-H | NA |
| V155 |  | 1-Palmitoylglycerol 3-phosphate | 0.803 | 0.005 | 1.786 | 409.236 | 1131.360 | M-H | Glycerophospholipid metabolism; Glycerolipid metabolism; Phosphatidylinositol signaling system |
| V156 |  | 2,3-Dihydro-5,5',7,7'-tetrahydroxy-2-(4-hydroxyphenyl)[3,8'-bi-4H-1-benzopyran]-4,4'-dione | 1.410 | 0.002 | 2.069 | 447.065 | 640.698 | M-H | NA |
| V157 |  | 7-Hydroxy-8-O-methylaloin B | 0.744 | 0.014 | 1.703 | 447.135 | 485.972 | M-H | NA |
| V158 |  | Buprenorphine | 1.212 | 0.026 | 1.359 | 466.294 | 526.588 | M-H | NA |
| V159 |  | Geranyl monophosphate | 0.746 | 0.011 | 1.453 | 467.192 | 353.116 | 2M-H | NA |
| V160 |  | 5-Formiminotetrahydrofolate | 1.205 | 0.032 | 1.306 | 471.171 | 263.206 | M-H | One carbon pool by folate |
| V161 |  | Rubraflavone D | 1.404 | 0.015 | 1.538 | 487.217 | 252.735 | M-H | NA |
| V162 |  | Alisol A | 0.820 | 0.015 | 1.449 | 489.360 | 663.797 | M-H | NA |
| V163 |  | (3b,4b,11b,14b)-11-Ethoxy-3,4-epoxy-14-hydroxy-12-cyathen-15-al 14-xyloside | 0.786 | 0.028 | 1.349 | 493.283 | 433.990 | M-H | NA |
| V164 |  | lysoPE 20:1 | 0.830 | 0.007 | 1.539 | 506.317 | 467.756 | M-H | Glycerophospholipid metabolism |
| V165 |  | 4-POHPA | 1.480 | 0.041 | 1.719 | 507.442 | 698.186 | M-H | NA |
| V166 |  | Leukotriene D4 methyl ester | 1.483 | 0.049 | 1.415 | 509.275 | 376.967 | M-H | NA |
| V167 |  | PE 22:1 | 0.781 | ＜0.001 | 2.005 | 548.337 | 485.486 | M-H | Glycerophospholipid metabolism; Glycosylphosphatidylinositol (GPI)-anchor biosynthesis |
| V168 |  | FAHFA 36:3 | 0.815 | 0.043 | 1.210 | 559.470 | 1050.790 | M-H | NA |
| V169 |  | MGDG 18:0 | 0.800 | 0.042 | 1.284 | 579.341 | 471.528 | M+FA-H | Glycerolipid metabolism |
| V170 |  | 10-Acetoxyligustroside | 1.266 | 0.044 | 1.352 | 581.185 | 252.756 | M-H | NA |
| V171 |  | Amphibine H | 1.464 | ＜0.001 | 2.568 | 604.318 | 292.357 | M-H | NA |
| V172 |  | Phosphatidylinositol lyso 20:4 | 1.230 | 0.006 | 1.653 | 619.291 | 1125.080 | M-H | NA |
| V173 |  | Neoacrimarine A | 1.323 | 0.002 | 1.894 | 680.284 | 309.399 | M-H | NA |
| V174 |  | MGDG 38:5 | 1.292 | 0.007 | 1.628 | 803.567 | 504.377 | M-H | Glycerolipid metabolism |
| V175 |  | 10-DEACETYL-7-XYLOTAXOL | 1.290 | 0.010 | 1.551 | 942.349 | 263.206 | M-H | NA |

Note: NA, Not Available; m/z, mass-to-charge ratio; Rt, retention time, FC, fold change; VIP, variable importance for the projection.

**Supplementary Table 5.**  **Mass spectrometry data of parent and product ions of potential biomarkers.**

| **Biomarkers** | **Parent ion** | | | | **Product ion** | |
| --- | --- | --- | --- | --- | --- | --- |
|  | **Scan mode** | **Rt (s)** | **Adducts** | **m/z** | **m/z** | **Intensity** |
| 3-β-D-Galactosyl-sn-glycerol | ESI- | 481.035 | M-H | 253.083 | 150.068 | 61 |
|  |  |  |  |  | 191.043 | 61 |
|  |  |  |  |  | 193.060 | 152 |
|  |  |  |  |  | 206.077 | 152 |
|  |  |  |  |  | 221.061 | 61 |
|  |  |  |  |  | 223.078 | 62 |
| γ-Aminobutyryl-lysine | ESI- | 545.151 | M-H | 230.155 | 83.942 | 10 |
|  |  |  |  |  | 84.016 | 10 |
|  |  |  |  |  | 100.932 | 30 |
|  |  |  |  |  | 126.954 | 15 |
|  |  |  |  |  | 127.077 | 10 |
|  |  |  |  |  | 145.011 | 15 |
|  |  |  |  |  | 212.101 | 8 |
| 7,8-Dihydroneopterin | ESI- | 481.173 | M-H | 254.086 | 59.024 | 15 |
|  |  |  |  |  | 71.013 | 15 |
|  |  |  |  |  | 147.066 | 15 |
|  |  |  |  |  | 162.052 | 15 |
|  |  |  |  |  | 164.063 | 15 |
|  |  |  |  |  | 165.067 | 15 |
|  |  |  |  |  | 166.096 | 15 |
|  |  |  |  |  | 192.047 | 15 |
|  |  |  |  |  | 222.066 | 30 |
| LPC 19:2 | ESI+ | 440.903 | M+Na | 556.346 | 86.098 | 42 |
|  |  |  |  |  | 104.108 | 42 |
|  |  |  |  |  | 125.000 | 42 |
|  |  |  |  |  | 184.076 | 582 |
|  |  |  |  |  | 522.262 | 146 |
| 6-Keto-prostaglandin F1α | ESI+ | 668.909 | M+H | 371.242 | 71.092 | 21 |
|  |  |  |  |  | 83.050 | 21 |
|  |  |  |  |  | 97.031 | 104 |
|  |  |  |  |  | 101.057 | 21 |
|  |  |  |  |  | 111.043 | 42 |
|  |  |  |  |  | 125.024 | 1255 |
|  |  |  |  |  | 129.048 | 21 |
|  |  |  |  |  | 139.041 | 167 |
|  |  |  |  |  | 167.032 | 723 |
|  |  |  |  |  | 241.098 | 21 |
|  |  |  |  |  | 265.031 | 21 |
|  |  |  |  |  | 281.051 | 21 |
| 17α,21-Dihydroxypregnenolone | ESI+ | 698.951 | M+H | 349.237 | 91.055 | 320 |
|  |  |  |  |  | 117.073 | 384 |
|  |  |  |  |  | 128.064 | 104 |
|  |  |  |  |  | 129.073 | 188 |
|  |  |  |  |  | 130.079 | 146 |
|  |  |  |  |  | 131.087 | 417 |
|  |  |  |  |  | 143.085 | 188 |
|  |  |  |  |  | 145.100 | 2402 |
|  |  |  |  |  | 155.084 | 313 |
|  |  |  |  |  | 157.062 | 44 |
|  |  |  |  |  | 171.115 | 42 |
|  |  |  |  |  | 173.095 | 4382 |
|  |  |  |  |  | 201.095 | 209 |
|  |  |  |  |  | 227.026 | 21 |
|  |  |  |  |  | 265.009 | 21 |
|  |  |  |  |  | 291.232 | 21 |
| γ-Glutamylphenylalanine | ESI- | 283.958 | M-H | 293.113 | 74.028 | 30 |
|  |  |  |  |  | 91.053 | 30 |
|  |  |  |  |  | 103.052 | 61 |
|  |  |  |  |  | 118.070 | 30 |
|  |  |  |  |  | 127.054 | 61 |
|  |  |  |  |  | 128.035 | 212 |
|  |  |  |  |  | 147.053 | 152 |
|  |  |  |  |  | 162.997 | 30 |
|  |  |  |  |  | 164.072 | 304 |
|  |  |  |  |  | 185.013 | 30 |
|  |  |  |  |  | 186.092 | 30 |
|  |  |  |  |  | 231.061 | 30 |
|  |  |  |  |  | 249.123 | 30 |
|  |  |  |  |  | 275.108 | 61 |

Note: Rt, retention time; m/z, mass-to-charge ratio; LPC, lysophosphatidylcholine.
